# Structural, Thermodynamic, and Kinetic Traits of Antiestrogen-Compounds Selectively Targeting the Y537S Mutant Estrogen Receptor α Transcriptional Activity in Breast Cancer Cell Lines

**DOI:** 10.3389/fchem.2019.00602

**Published:** 2019-09-04

**Authors:** Matic Pavlin, Luca Gelsomino, Ines Barone, Angelo Spinello, Stefania Catalano, Sebastiano Andò, Alessandra Magistrato

**Affiliations:** ^1^National Research Council – Institute of Materials (IOM) at International School for Advanced Studies (ISAS), Scuola Internazionale Superiore di Studi Avanzati (SISSA), Trieste, Italy; ^2^Department of Pharmacy, Health and Nutrition Sciences, Centro Sanitario, University of Calabria, Rende, Italy

**Keywords:** estrogen receptor, breast cancer, SERM, SERD, molecular dynamics, Y537S, resistant breast cancers

## Abstract

The most frequently diagnosed cancers in women are the estrogen receptor (ER)-positive breast cancer subtypes, which are characterized by estrogen dependency for their growth. The mainstay of clinical treatment for this tumor relies on the modulation of ERα action or on the suppression of estrogen biosynthesis via the administration of Selective ERα Modulators/Down-regulators (SERMs/SERDs) or aromatase inhibitors, respectively. Nevertheless, *de novo* and acquired resistance to these therapies frequently occurs and represents a major clinical concern for patient survival. Recently, somatic mutations affecting the hormone-binding domain of ERα (i.e., Y537S, Y537N, D538G) have been associated with endocrine resistance, disease relapse and increased mortality rates. Hence, devising novel therapies against these ERα isoforms represents a daunting challenge. Here, we identified five molecules active on recurrent Y537S ERα polymorphism by employing *in silico* virtual screening on commercial databases of molecules, complemented by ER-transactivation and MTT assays in MCF7 and MDA-MB-231 breast cancer cells expressing wild type or mutated ERα. Among them, one molecule selectively targets Y537S ERα without inducing any cytotoxicity in breast cell lines. Multi-microseconds (4.5 μs) of biased and unbiased molecular dynamics provided an atomic-level picture of the structural, thermodynamics (i.e., binding free energies) and the kinetic (i.e., dissociation free energy barriers) of these active ligands as compared to clinically used SERM/SERDs upon binding to wild type and distinct ERα variants (Y537S, Y537N, D538G). This study contributes to a dissection of the key molecular traits needed by drug-candidates to hamper the agonist (active)-like conformation of ERα, normally selected by those polymorphic variants. This information can be useful to discover mutant specific drug-candidates, enabling to move a step forward toward tailored approaches for breast cancer treatment.

## Introduction

**Figure 1:**
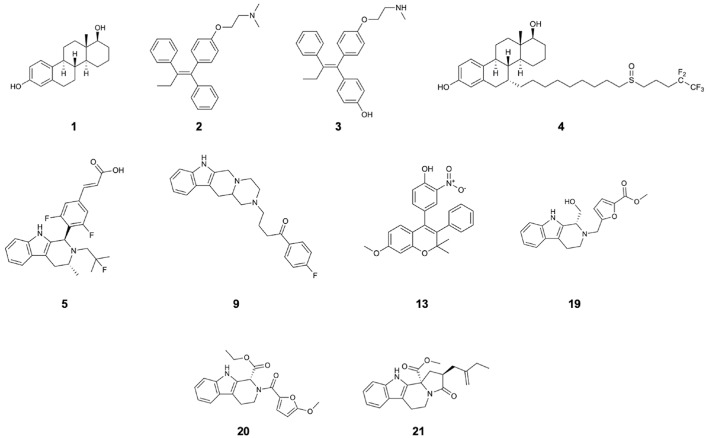
Structures of 17β-estradiol (**1**), tamoxifen (**2**), endoxifen (**3**), fulvestrant (**4**), AZD-9496 (**5**), and compounds **9**, **13**, **19**, **20**, **21** which are the identified drug-candidates active against Y537S ERα.

Breast Cancer (BC) is the most frequent cancer type and the second leading cause of death in women, representing 25% of all cancers. In ~70% of the BC cases detected after the menopause, cellular proliferation is mediated by estrogens (**1**; Figure 1) binding to their specific nuclear hormone receptor [Estrogen Receptor α (ERα, *ESR1*)] (Fanning and Greene, 2019).

This latter is a ligand-activated transcription factor, which upon estrogen binding, decreases apoptosis and/or promotes cell proliferation, ultimately playing a pro-oncogenic role. Hence, in the most diffused BC type cell proliferation relies on the expression of ERα, and on the presence of blood circulating estrogens, being hence classified as ER sensitive (ER+). Gold standard endocrine treatments against ER+ BC consist in suppressing estrogen biosynthesis, via the administration of aromatase inhibitors, or in counteracting ERα pro-oncogenic action via the drugging of selective ERα modulators (SERMs) or downregulators (SERDs). Namely, SERMs [tamoxifen and its most abundant metabolite endoxifen (END)] act as antagonists, occupying the estrogen binding site and inducing a conformational change of ERα toward an inactive conformation. SERDs [fulvestrant (FULV)], instead, also foster ERα ubiquitination and degradation (Fanning et al., 2016; Pavlin et al., 2018).

Similarly to other nuclear receptors, ERα presents a puzzling tridimensional structure which atomic-level organization remains controversial (Huang et al., 2018). This is composed out of five distinct functional domains (Supplementary Figure 1), among which only the structures of the DNA-binding domain and the ligand-binding domain (LBD) have been characterized. The LBD is active as a homodimer with each monomer hosting a ligand binding cavity (LBC). The LBD crystal structures (Supplementary Figure 2) revealed that upon binding of an agonist or an antagonist molecule, helix 12 (H12) can undergo a conformational switch between the active and inactive form, respectively (Joseph et al., 2016). In the agonist (active) state, H12 faces helices H3, H5/6, and H11, closing the LBC (Supplementary Figure 2A; Robinson et al., 2013). Conversely, in the antagonist (inactive) form, H12 rearranges, as a consequence of SERM/SERD-binding, moving toward the groove lined by H3 and H5 (Supplementary Figure 2B; van Kruchten et al., 2015; Joseph et al., 2016).

In the last decades, SERMs have been proved to be highly beneficial, substantially decreasing the mortality rates of woman affected by BC cancer type by 25–30 %. The most effective ERα antagonists in clinical use are: (i) tamoxifen (**2**; Figure 1), a SERM, which in spite of its beneficial action in breast tissues, is plagued by agonistic effects in peripheral ones (endometrium), and is active through its metabolite, endoxifen (**3**; Figure 1), and (ii) FULV (**4**; Figure 1), a potent SERD (Nilsson and Gustafsson, 2011; van Kruchten et al., 2015) characterized, however, by poor solubility. This makes its administration arduous and therefore probably limiting its efficacy. These adjuvant therapies are administered over extended time frame (5–10 years) to control tumor growth, or, even, to prevent disease in case of BC-prone genetic profiles. Nevertheless, after prolonged exposure to these therapies, tumors evolve by adapting to the pharmacological pressure. Distinct studies highlighted a stunningly complex, composite, and multifactorial genomic landscape as responsible of tumor refractoriness to treatments (Spinello et al., 2019b). This has mostly been associated to an alteration of mitogen activated kinase pathway (MAPK) or of a deregulated estrogen receptor transcriptional activity (Razavi et al., 2018). This latter takes place when ERα acquires new structural traits, eventually leading to resistance and relapse to therapies. Scaringly, almost 50 % of all ER+ BC patients, initially benefiting from first-line therapy, will eventually develop resistance after prolonged treatments (acquired resistance). This ultimately results in a shortening of their survival time.

It is nowadays well-established (Merenbakh-Lamin et al., 2013; Robinson et al., 2013; Toy et al., 2013; Jeselsohn et al., 2014) that distinct ERα polymorphisms (mERαs), located in the vicinity of LBC (i.e., E380Q), between H9 and H10, or in the loop connecting H11 and H12 (i.e., L536Q, L536R, Y537C, Y537N, Y537S, and D538G), are recurrently observed in metastatic BC patients relapsing after extended treatments regimens. The most abundant ERα polymorphisms observed in LBD are D538G (occurrence of 21–36 % of cases (Jeselsohn et al., 2014; Chandarlapaty et al., 2016; Toy et al., 2017), Y537N (5–33 %) (Jeselsohn et al., 2014; Toy et al., 2017), and Y537S (13–22 %) (Jeselsohn et al., 2014; Chandarlapaty et al., 2016; Toy et al., 2017). This latter remains the most aggressive isoform (survival rate of 20 months as compared to 26 of D538G) (Chandarlapaty et al., 2016). Most of these mutations are insensitive to tamoxifen, while still responding to FULV. As such, tumors proliferation still depends on ERα expression, underlying the still unmet oncological need of a complete inhibition/abrogation of its signaling pathway (Busonero et al., 2019).

Targeted therapy counteracting mERα is a current object of intense preclinical and early clinical interest, as also evidenced by the significant number of studies aiming at identifying orally bioavailable SERDs, eventually able to overcome resistance (De Savi et al., 2015; Fanning et al., 2018; Hamilton et al., 2018; Sharma et al., 2018; Kahraman et al., 2019; Scott et al., 2019). Among these GDC-810, AZD9496 (**5**; Figure 1) (hereafter AZD, a drug in preclinical use as oral SERD, for which a first clinical study have been recently accomplished) (Weir et al., 2016; Hamilton et al., 2018), and LSZ102 have been identified (Scott et al., 2019).

Aiming at selectively targeting specific and aggressive ERα variants, we have recently meticulously annotated the structural and dynamical alterations induced to ERα structure by each recurrent polymorphism, disclosing that each of them triggers the acquisition of a different agonist-like (intrinsically active) conformation of H12. As a result, tumors bearing these isoforms proliferate irrespectively of estrogen production, with SERMs and aromatase inhibitors' efficacy being lost after their appearance (Spinello et al., 2019b). Since these ER+ BC cells are sensitive to FULV and AZD, we also performed extensive MD simulations typifying the structural features responsible of distinct efficacy of FULV and AZD on mutant (m)ERα, as opposite to END.

Stunningly, our computational assays disclosed that the FULV and AZD's elongated shape, owing to their aliphatic and carboxylic moiety, respectively, was the key structural determinant counteracting the acquisition of an H12 agonist-like conformation (Fanning et al., 2016; Pavlin et al., 2018). Understanding the structural signature needed by drugs able to effectively fight metastatic and refractory BC types was a necessary prerequisite for the rational discovery of mutant-specific SERMs/SERDs. Building on this knowledge, here, we performed virtual screenings on existing database of molecules, and we tested their efficacy on MCF7 and MDA-MB-231 cell lines harboring Wild Type (WT) and Y537S, D538G, and Y537N ERα variants. As a result, we identified five molecules able to counteract the enhanced transcriptional activity of Y537S ERα isoform, with one being non-cytotoxic and preferentially active toward the Y537S variant. The structural and dynamical impact of the five active molecules, as well as their thermodynamic and kinetic properties (of the best molecule) were also explored via biased and unbiased classical Molecular Dynamics (MD) simulations, and compared with those of END, AZD, and FULV in order to dissect the source of their distinct efficacy profiles. These outcomes may lead toward the discovery of isoform-selective drug-candidates, providing a therapeutic option for the specific genomic profile of ER+ BC patients relapsing mainstay therapies.

## Materials and Methods

### *In silico* Screening

The NCI (https://cactus.nci.nih.gov/download/nci/) library Release 4 Files Series—May 2012 (containing 265,242 structures) was used for virtual screening (VS) studies. Compounds were filtered using the Schrodinger Suite 2017-1 Ligfilter tool (2017). In order to eliminate molecules possessing poor absorption and permeation we applied Lipinski's rule of five (Lipinski et al., 2001). Further, filtering was applied to compounds bearing more than 10 rotatable bonds, since high ligand flexibility implies higher entropic contributions and reduces oral availability (Veber et al., 2002). Next, QikProp (2017) was employed to predict LogP values of the compounds to assess information on their solubility in water.

After ligand preparation *in silico* screening of the library was performed on different mERα structures. Namely, in order to account for receptor's flexibility in the screening we considered five different ERα conformations as target structures (Pavlin et al., 2018). These were selected from the populated cluster extracted from 500 ns-long classical MD simulations trajectories of AZD and FULV in complex with the Y537S, Y537N, and D538G isoforms obtained in our previous study (Pavlin et al., 2018). In this respect, we employed two conformations for Y537S ERα (in complex with AZD and FULV), one for Y537N (in complex with FULV, since conformation of this mutant complex with AZD was similar to that of Y537S), and two structures for D538G (in complex with AZD and FULV). A van der Waals (vdW) radius scaling factor of 0.80 Å for protein and ligands atoms having a partial charge < 0.15 was used to account for protein flexibility. Size of the box for *in silico* screenings was determined by considering the residues interacting with different antagonists placed inside the binding site and those residues pinpointed as critical for antagonizing ERα activation in our previous work (Pavlin et al., 2018).

In order to obtain a set of promising ligands for experimental testing, we followed two protocols of VS. First, a workflow based on three subsequent steps of docking with increasing level of accuracy for each ERα conformation, was adopted by using the Glide program (Friesner et al., 2004). Namely, (i) a fast high-throughput virtual screening (HTVS) was initially performed in order to efficiently select promising ligands among ~220,000 of compounds from the pre-filtered NCI library; (ii) 10 % of the best ranked ligands (~22,000 structures per each ERα conformation) were retained and a single precision (SP) docking calculation was done; (iii) the top 10 % of the resulting compounds (~2,200 structures per each ERα conformation) were screened using the extra precision (XP) protocol. This latter should eliminate false positives by using a more extensive sampling and more accurate scoring functions. END, AZD, and FULV were also docked to assess the quality of our results as reported in Supplementary Table 1. The molecules resulting from the screening were sorted according to GlideScore scoring function. The selection criterion for further investigation was that the screened compounds had docking score lower than −8.5 kcal/mol and that displayed favorable interactions with at least one of the five mERαs target structures (i.e., two structures extracted from the MD trajectory of Y537S ERα in complex with AZD and FULV, one for Y537N in complex with FULV, and two structures for D538G in complex with AZD and FULV). This was done in order to find a good compromise between the number of molecules selected for experimental screening and the quality of the docking score. Moreover, our reference molecules FULV and AZD exhibit on the target structure the same range of docking score values.

In the second protocol, we initially performed ligand-based screening using the CANVAS program (Duan et al., 2010; Sastry et al., 2010). Here the searching criteria were based on the scaffold that antagonist should possess. The latter was defined considering the common structural features that an effective SERD should have according to our previous study (Pavlin et al., 2018), more precisely, selected ligand should have scaffold based either on the END scaffold or on the tri-membered ring scaffold of AZD in order to stabilize ligand inside LBC, together with a polar tail that is able to form hydrogen bonds (H-bonds) with the H11-12 loop. The 415 selected ligands were then screened to all five mERαs conformations by using XP protocol. In this second case, the cut-off docking score for selection of ligands was −7.5 kcal/mol, and the ligands were selected only when displaying favorable interactions with at least two distinct mERα structures among the five target structures used and at least one was within the cut-off range. These molecules were available as donation of the National Cancer Institute USA. Other known activities of the five molecules observed to be active on Y537S in this study are reported in Supplementary Table 7.

### Reagents, Antibodies, and Plasmids

17β-estradiol was purchased from Sigma (St. Louis, MO, USA). Antibodies against ERα and GAPDH were from Santa Cruz Biotechnology (Santa Cruz, CA, USA). Yellow fluorescent protein (YFP)-tagged expression constructs, YFP-WT, YFP-Y537S, YFP-Y537N and YFP-D538G ERα were generated as previously described (Gelsomino et al., 2016, 2018) XETL plasmid, containing an estrogen-responsive element, was provided by Dr. Picard (University of Geneva, Geneva, Switzerland).

### Cell Cultures

Human MCF-7 and MDA-MB-231 BC cells were acquired in 2015 from American Type Culture Collection, stored and cultured according to supplier's instructions. Cells were used within six-months after frozen-aliquot resuscitations and regularly tested for Mycoplasma-negativity (MycoAlert, Lonza, Basilea, Switzerland).

### Immunoblot Analysis

Equal amounts of proteins were resolved on 10% SDS-PAGE as previously described (Giordano et al., 2016). The antigen-antibody complex was revealed using the ECL System (Bio-rad, Hercules, CA, USA). Images were acquired using Odissey FC from Licor (Lincoln, Nebraska, USA). Blots are representative of three independent experiments.

### ERα Transactivation Assay

ERα transactivation assay was performed as previously reported (Barone et al., 2011). Briefly, MCF-7 and MDA-MB-231 cells (50,000/well) were plated in phenol red-free with 5 % charcoal-stripped FBS in 24-well plates. After 24 h, cells were co-transfected with 0.5 μg of reporter plasmid XETL plus 0.1 μg of YFP-tagged expression constructs and 20 ng of TK Renilla luciferase plasmid as an internal control. Transfection was performed using the Lipofectamine 2000 reagent (Life Technologies, Carlsbad, CA, USA) as recommended by the manufacturer. Six hours after transfection, the medium was changed and the cells were treated as indicated for 24 h. Firefly and Renilla luciferase activities were measured using a Dual Luciferase kit (Promega, Madison, WI, USA). The firefly luciferase data for each sample were normalized on the basis of transfection efficiency measured by Renilla luciferase activity (Rizza et al., 2014). Data represent three independent experiments, carried out in triplicate.

### MTT Cell Viability Assay

1,000 cells were plated into 96-well plates in phenol red-free medium containing 5 % charcoal-stripped FBS. After 24 h, cells were exposed to the different treatments as indicated. One day later, cell viability was assessed by (3-(4,5-Dimethylthiazol-2-yl)-2,5-Diphenyltetrazolium-Bromide) (MTT, Sigma-Aldrich) as described (Covington et al., 2013). Results are expressed as fold change relative to vehicle-treated cells. Data represent three-independent experiments, performed in triplicate.

### Real-Time RT-PCR Assays

Total RNA was extracted from cells using TRIzol reagent (Life Technologies). Purity and integrity of the RNA were confirmed spectroscopically and by gel electrophoresis before use. One microgram of total RNA was reverse transcribed in a final volume of 20 μL using the RETROscript kit (Life Technologies) and cDNA was diluted 1:3 in nuclease-free water. The evaluation of *TFF1, CTSD, CCND1* and *MYC* mRNA expression was performed by real-time RT-PCR, using SYBR Green Universal PCR Master Mix (Bio-rad). The relative gene expression levels were calculated using the ΔΔCt method as described (Catalano et al., 2015). Primers are listed in Supplementary Table 2.

### Statistical Analysis of Experimental Data

Data were analyzed for statistical significance using two-tailed student's Test using GraphPad-Prism5 (GraphPad Software, Inc., San Diego, CA). Standard deviations (S. D.) are shown.

### Classical MD Simulations

Of the 17 tested molecules (Figure 1, Supplementary Figure 3) obtained by both protocols of VS (four from the structure-based strategy and the rest by the ligand-based strategy as reported in Supplementary Table 1) five resulted to be active in the *in vitro* tests. These latter were also docked to the WT ERα using the XP protocol. All active molecules were subjected to MD simulations in complex with the WT and Y537S ERα variants. Additionally, compound **19**, showing the most promising results in experimental tests, was also docked and simulated in complex with Y537N and D538G ERα.

Physiological protonation states of ERα were already determined previously (Pavlin et al., 2018) using the webserver H++ (Anandakrishnan et al., 2012). Parm99SB AMBER force field (FF) with ILDN modification was employed for the protein (Wickstrom et al., 2009; Lindorff-Larsen et al., 2010), and the general Amber FF (GAFF) (Wang et al., 2004) was used for ligands. ESP charges (Bayly et al., 1993) were calculated by performing geometry optimizations of the ligands at Hartree-Fock level of theory using a 6-31G^*^ basis set with the Gaussian 09 software (Frisch et al., 2016) and were later transformed in RESP charges with the Antechamber module of Ambertools16 (Wang et al., 2006). Since the dockings were performed on monomers, while the LBD in physiological conditions is a dimer, we built each dimer by superimposing the monomer on each of the two dimers of the corresponding mERα conformation from our previous work (Pavlin et al., 2018).

Each system was solvated using TIP3P waters (Jorgensen et al., 1983) in a truncated octahedron box with minimum distance of 12 Å between solute and the edge of the box, leading to a total of ~95,000 atoms. MD simulations were performed with GROMACS 5.0.4 (Abraham et al., 2015). An integration time step of 2 fs was used and all covalent bonds involving hydrogen atoms constrained with the LINCS algorithm (Hess et al., 1997). Particle Mesh Ewald algorithm (Darden et al., 1993) was used in order to account electrostatic interactions. Simulations were performed in the isothermal-isobaric NPT ensemble, at a temperature of 310 K, under control of a velocity-rescaling thermostat (Bussi et al., 2007). Preliminary energy minimization was done with the steepest descend algorithm. Next, all systems were heated to the final temperature of 310 K using 40 steps of simulated annealing (0-90 K in steps of 5 K/25 ps; 90-310 K in steps of 10 K/25 ps). WT ERα models underwent 300 ns long classical MD simulations (last 200 ns were used for analysis), while mERα ones underwent 400 ns long simulations and last 300 ns were used for further analysis.

### Metadynamics

In order to further refine the binding poses and better dissect the impact of the kinetic properties on efficacy and selectivity, we performed FF-based Metadynamics (MTD) simulations of AZD and **19**. In particular, MTD runs of 60–130 ns were done to refine the binding pose and study ligand dissociation with GROMACS 5.0.4 using the PLUMED 2.0 plugin (Tribello et al., 2014). Two collective variables (CVs) were used: the first CV (CV1) describes the number of either hydrogen bonds (AZD) or hydrophobic contacts (**19**) between the ligands and the LBC, computed as a coordination number; the second CV (CV2) corresponds to the distance between the center of masses (COM) of the protein and the ligand. Gaussian hills having a height of 0.6 kJ/mol in all systems, while the widths of 0.06 and 0.015 (AZD), 0.40 and 0.025 (**19**), were added, respectively, for CV1 and CV2 every 4 ps of MD. A harmonic wall was used to restrain the exploration of the FES on CV2 at the value of 3.5 nm. Three replicas of the MTD simulations were performed, starting from different frames as extracted from the equilibrated MD trajectory and the uncertainty of the dissociation free energy barriers (Δ${\text{G}}_{\text{b}}^{\text{\#}}$) were estimated from the standard deviation of the barriers obtained out of the three replicas, following a protocol adopted in previous studies (Bisha et al., 2013; Sgrignani and Magistrato, 2015; Spinello et al., 2018, 2019a).

### Simulation Analysis

Cluster analysis and root mean square deviation (RMSD) of the MD trajectories were done with the g_cluster tool, based on the Daura et al algorithm (Daura et al., 1999), and g_rms, as implemented in the GROMACS 5.0.4 program (Abraham et al., 2015). Molecular Mechanics Generalized Born Surface Area (MM-GBSA) free energy calculation were performed with the MM_PBSA.py tool of Amber 18 program, following a procedure successfully applied in previous studies (Spinello et al., 2019a,c). Visualization of the MD trajectories was done with the VMD program (Humphrey et al., 1996), while the images were prepared using UCSF Chimera1.12 visualization tool (Pettersen et al., 2004).

#### Correlation Analysis

The covariance matrices were constructed from the atoms position vectors upon an RMS-fit to the starting configuration of the MD run as to remove the rotational and translational motions. Each element in the covariance matrix is the covariance between atoms i and j, defining the i, j position of the matrix. The covariance C_ij_ is defined as

(1) $$C_{ij}=\langle \left({\vec{r}}_{\;i}-\langle {\vec{r}}_{\;i}\rangle \right)\left({\vec{r}}_{\;j}-\langle {\vec{r}}_{\;j}\rangle \right)\rangle ,$$

where ${\vec{r}}_{i}$ and ${\vec{r}}_{j}\;$are the position vectors of atoms i and j, and the brackets denote an average over the sampled time period. The diagonalization of the covariance matrix leads to a set of orthogonal collective eigenvectors, each associated to a corresponding eigenvalue. The eigenvalues denote how much each eigenvector is representative of the system dynamics.

The cross-correlation matrices (or normalized covariance matrices) based on the Pearson's correlation coefficients (CC_ij_) were calculated with the *cpptraj* module of Ambertools 18 from the calculated covariance matrices. Each element of the cross-correlation matrix in the i,j position corresponds to a Pearson's CC_ij_, i.e., the normalized covariance between atoms i and j calculated with the formula:

(2) $$C_{ij}=\;\frac{\langle \left({\vec{r}}_{i}-\langle {\vec{r}}_{i}\rangle \right)\left({\vec{r}}_{j}-\langle {\vec{r}}_{j}\rangle \right)\rangle }{\left[\left(\langle {\vec{r}}_{i}^{2}\rangle -{\langle {\vec{r}}_{i}\rangle }^{2}\right)\left(\langle {\vec{r}}_{j}^{2}\rangle -{\langle {\vec{r}}_{j}\rangle }^{2}\right)\right]},$$

here the normalization factor is the product between the standard deviations of the two position vectors. As a result, CC_ij_ range from a value of −1, for a totally negatively correlated motion between two atoms, and a value of +1, which instead means a positively correlated lockstep motion. Here we have also calculated the correlation scores (CSs) between each LBD helix and all the others, dividing each as depicted in Supplementary Figure 2 (Pavlin et al., 2018). Then, we calculated the sum of CC_ij_ between the residues i belonging to the helix I and the residues j belonging to the helix J. Importantly, the values −0.6 < CC_ij_ < +0.6 were discarded in order to eliminate the noise due to uncorrelated motions (Palermo et al., 2016; Casalino et al., 2018) and the sum of the cross-correlation score was divided by the product of the number of residues contributing to the score as a correlation density.

## Results

### *In silico* Screening and *in vitro* Studies

Building on our previous classification of structural traits of drugs effectively targeting mERα (Pavlin et al., 2018), we performed *in silico* screening on the structures obtained from MD simulations of Y357S, Y537N, and D538G mutants hosting AZD and FULV in the LBC. A detailed list of the molecules tested as well as their docking score on each specific target structure is reported in Supplementary Figure 3 and Supplementary Table 1, respectively. Remarkably our newly developed VS strategy allows not only to account for the distinct conformations that the receptor can adopt at finite temperature, as in the ensemble docking, but it also encompasses the induced fit effects exerted by the binding of efficacious drugs to distinct ERα isoforms (Spinello et al., 2019a). The best-ranked 17 compounds (**6**-**22**), that were binding to more than one ERα isoform in VS, were then experimentally tested.

**Figure 2:**
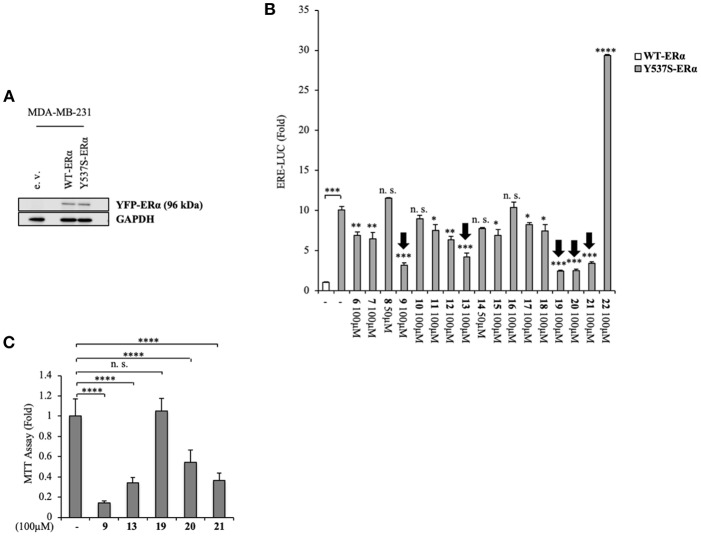
Inhibition of Y537S ERα activation exerted by the compound **19** in MDA-MB-231 breast cancer cells. **(A)** Immunoblotting showing exogenous ERα protein expression in ERα-negative MDA-MB-231 breast cancer cells transiently transfected with an empty vector (e. v.), YFP-WT or YFP-Y537S ERα expression vectors. GAPDH was used as a control for equal loading and transfer. **(B)** ERα-transactivation assay in MDA-MB-231 cells transiently transfected with YFP-WT or YFP-Y537S ERα expressing vectors plus an ERE-luciferase reporter (XETL), and treated with vehicle (-) or the different compounds (**6**-**22**), as indicated. Data are reported as fold change relative to WT-ERα expressing cells. **(C)** MTT cell viability assay in MDA-MB-231 cells treated with vehicle (-) or the different compounds (**9**, **13**, **19**, **20**, **21**, 100 μM), as indicated for 24 h. Results are expressed as fold change relative to vehicle-treated cells. The values represent the mean ± S. D. of three different experiments, each performed in triplicate. n. s., non-significant; ^*^P < 0.05; ^**^P < 0.005; ^***^P < 0.0005; ^****^P < 0.00005.

Namely, their effect on the transcriptional activity of the mutant Y537S ERα was investigated in cell-based assays, using a standard genomic transcriptional output method (i.e., the estrogen response element (ERE) - luciferase-based gene transactivation system) for assessing their ability to bind ERα and, subsequently, transactivate an ERE-mediated transcription, allowing an assessment of the transcriptional responses of each receptor separately. Thus, human ERα-negative MDA-MB-231 BC cells were cotransfected with either YFP-WT or YFP-Y537S ERα expression vectors along with an ERE-luciferase reporter plasmid (XETL) and treated with the vehicle or the selected compounds (**6**-**22**). As shown in Figure 2A, cells expressed similar levels of the 96 kDa protein representing the exogenously added WT or Y537S mutant receptor tagged with YFP. In line with previous results (Toy et al., 2013), reporter gene transactivation assays showed that control basal activity of Y537S ERα was more elevated than that of WT (Figure 2B). Importantly, the tested compounds exerted different effects on Y537S ERα transcriptional activity, with **9**, **13**, **19**, **20**, and **21** showing the highest efficacy in reducing the activity of the Y537S mutant (76–57 % decrease) when used at 100 μM concentration. Hence, these were chosen to evaluate their potential toxicity in MDA-MB-231 cells by using MTT cell survival assay (Figure 2C). As a result, compounds **9**, **13**, **20**, and **21** markedly reduced cell viability even in MDA-MB-231 cells, whereas the compound **19** did not provoke any significant effects at the dose tested. Thus, among these compounds, **19** represents the best-candidate for further studies. Among these compounds, **9**, **19**, **20**, and **21** share the same chemo-type of the parent compounds AZD.

MDA-MB-231 BC cells transfected with Y537S-ERα vectors and treated with compound **19** at increasing doses (from 1 nM to 100 μM) displayed a dose-dependent decrease of Y537S-ERα transactivation, with the highest inhibition registered at 100 μM concentration (65 ± 10 % inhibition as compared to vehicle) (Supplementary Figure 4).

**Figure 3:**
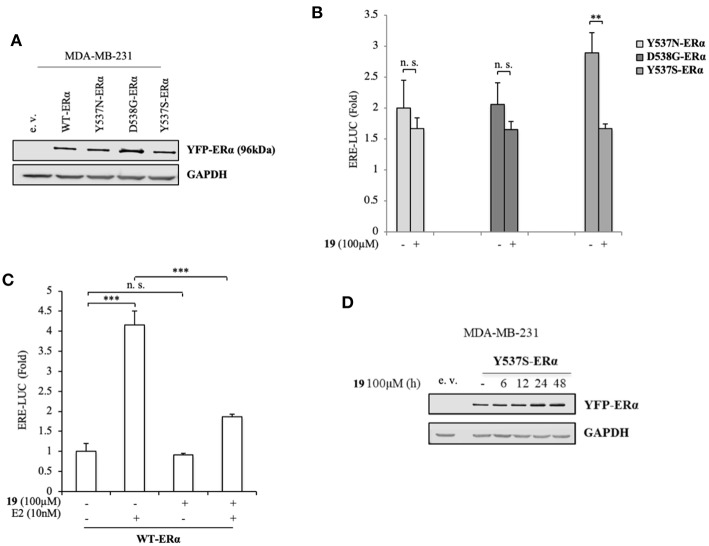
Impact of compound **19** on activation of WT, Y537N, and D538G ERα in MDA-MB-231 breast cancer cells. **(A)** Immunoblotting showing exogenous ERα protein expression in ERα-negative MDA-MB-231 breast cancer cells transiently transfected with an empty vector (e. v.), YFP-WT, YFP-Y537N, YFP-Y537S, or YFP-D538G ERα expressing vectors. GAPDH was used as a control for equal loading and transfer. **(B)** ERα-transactivation assay in MDA-MB-231 cells transiently transfected with YFP-Y537N, YFP-Y537S, or YFP-D538G ERα expressing vectors plus an ERE-luciferase reporter (XETL), and treated with vehicle (-) or the compound **19** at 100 μM. Data are reported as fold change relative to WT-ERα expressing cells. **(C)** ERα-transactivation assay in MDA-MB-231 cells transiently transfected with YFP-WT plus XETL plasmid, and treated with vehicle (-) or the compound **19** (100 μM) in the presence or not of 17β-estradiol (E2, 10 nM). Data are reported as fold change relative to vehicle (-)-treated cells. The values represent the mean ± S. D. of three different experiments, each performed in triplicate. n. s., non-significant; ^**^P < 0.005; ^***^P < 0.0005. **(D)** Immunoblotting showing ERα protein expression in MDA-MB-231 breast cancer cells transiently transfected with YFP-Y537S ERα expressing vector and treated with the compound **19** (100 μM) at the indicated time. GAPDH was used as a control for equal loading and transfer.

To better clarify the activity of compound **19**, we also evaluated its ability to affect ERα transactivation in cells expressing other two frequently-occurring mutations: YFP-Y537N and YFP-D538G ERα mutations (Figure 3A). Surprisingly, **19** has a smaller effect in hampering the transactivation of these mutants (Figure 3B), stunningly pinpointing its selectivity in antagonizing preferentially the transcriptional activity of the Y537S ERα isoform.

Next, we inspected its effects on YFP-WT ERα expressing cells in the presence/absence of 17β-estradiol (E2), the endogenous ERα ligand (Figure 3C). As expected, E2 treatment was able to trigger luciferase expression through the ERE interaction. Notably its treatment with compound **19**, while not significantly altering WT-ERα transactivation, was associated with a drastic reduction in E2-mediated effects. This suggests that ligand **19** may compete with E2 for the LBC. Interestingly, treatment with **19** was not associated with a down-regulation of Y537S ERα levels (Figure 3D).

**Figure 4:**
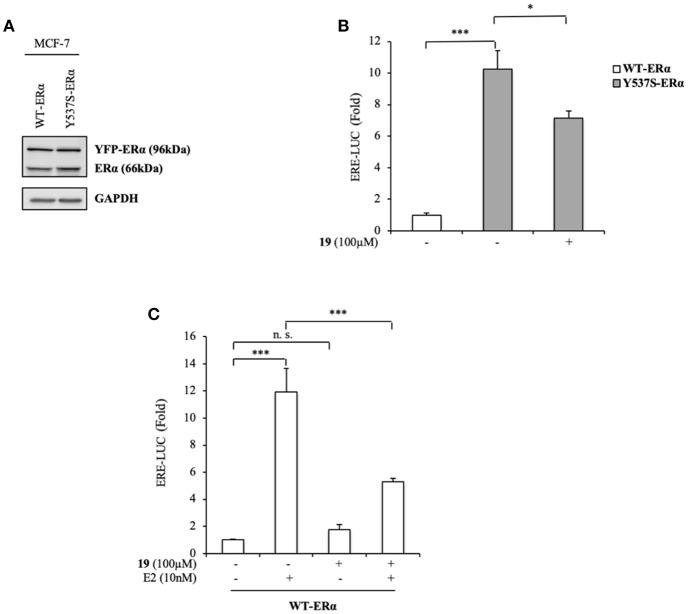
Effects of compound **19** on WT and Y537S ERα activation in MCF-7 breast cancer cells. **(A)** Immunoblotting showing exogenous and endogenous ERα protein expression in ERα-positive MCF-7 breast cancer cells transiently transfected with YFP-WT or YFP-Y537S ERα expressing vectors. GAPDH was used as a control for equal loading and transfer. **(B)** ERα-transactivation assay in MCF-7 cells transiently transfected with YFP-WT or YFP-Y537S ERα expressing vectors plus an ERE-luciferase reporter (XETL), and treated with vehicle (-) or the compound **19** at 100 μM, as indicated. Data are reported as fold change relative to WT-ERα expressing cells. **(C)** ERα-transactivation assay in MCF-7 cells transiently transfected with YFP-WT plus XETL plasmid, and treated with vehicle (-) or the compound **19** (100 μM) in the presence or not of 17β-estradiol (E2, 10 nM). Data are reported as fold change relative to vehicle (-)-treated cells. The values represent the mean ± S. D. of three different experiments, each performed in triplicate. n. s., non-significant; ^*^P < 0.05; ^***^P < 0.0005.

To expand our investigation, the potency of the compound **19** in affecting Y537S ERα activity was also tested in ER+ MCF-7 BC cells bearing the YFP-WT and YFP-Y537S receptor. These expressed a 66 kDa endogenous ERα, along with a 96 kDa receptor represent the exogenously added WT and mERα tagged with YFP (Figure 4A). As previously shown for MDA-MB-231 BC cells, we found a significant increase of YFP-Y537S receptor transcriptional activity as compared to that of YFP-WT ERα and this induction was reduced upon exposure to compound **19** (Figure 4B). In addition, **19** antagonized E2-mediated effects also in YFP-WT ERα MCF-7 expressing cells, without exerting any action on basal WT ERα activity (Figure 4C).

**Figure 5:**
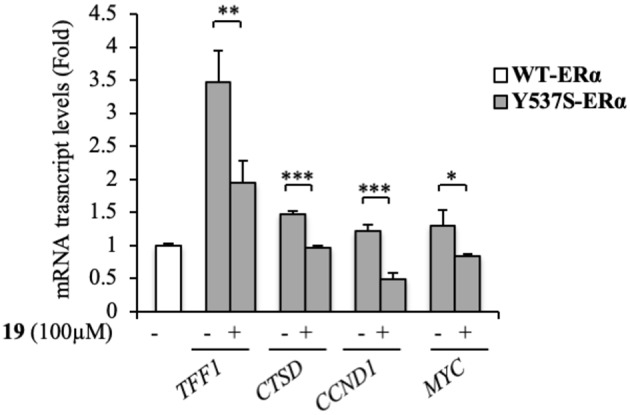
Effects of compound **19** on ERα target genes in YFP-Y537S ERα expressing MDA-MB-231 breast cancer cells. Real time RT-PCR assay for Trefoil Factor 1 (*TTF1*), cathepsin D (*CTSD*), cyclin D1 (*CCND1*) and c-Myc (*MYC*) mRNA expression. The values represent the mean ± S. D. of three different experiments, each performed in triplicate. n. s., non-significant; ^*^P < 0.05; ^**^P < 0.005; ^***^P < 0.0005.

At a molecular level, ERα activation and association with the ERE result in an enhanced expression profiles of a number of downstream target genes, including those for trefoil factor 1/pS2, cathepsin D, cyclin D1, and c-Myc (Barone et al., 2010). The biological correlation of the inhibition of Y537S ERα transactivation induced by **19** is the down-regulation of the classical estrogen-regulated genes in MDA-MB-231 cells (Figure 5), confirming the binding of this antagonist to ERα, well-fitting with *in silico* predictions.

### Atomic-Level Understanding of Drugs Efficacy

In order to identify the structural and dynamics features responsible of the efficacy and the selectivity of compound **19** toward Y537S ERα, while being inefficacious and/or displaying limited efficacy on WT, D538G and Y357N, we performed extensive MD simulations of the five active molecules in complex with the WT and Y537S ERα isoforms, starting from binding poses obtained from docking simulations.

**Figure 6:**
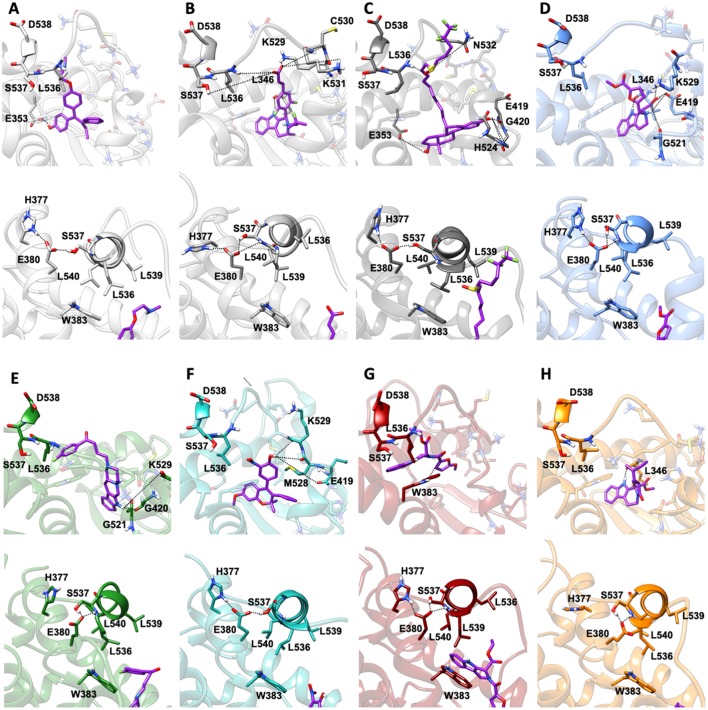
Binding of the five active molecules to Y537S ERα as compared with END, AZD, and FULV. **(A)** END; **(B)** AZD; **(C)** FULV; **(D)** compound **19**; **(E) 9**; **(F) 13**; **(G) 20**; **(H) 21**. Top panels show their placement in the ligand binding cavity, while bottom panels display a close view of E380 H-bond network induced by each ligand. Inhibitors are shown in licorice with carbon atoms in purple color, while oxygen and nitrogen are shown in red and blue, respectively. Protein is depicted in gray new cartoons for END, AZD, FULV, and blue, green, light green purple orange new cartoons for **19**, **9**, **13**, **20**, **21**, respectively.

MD simulations revealed two important and common structural traits among the inspected compounds, also shared by AZD and FULV. All molecules occupy the binding cavity protruding toward the H11-12 loop, which hosts the Y537S variant (Figure 6). Three of them (**9**, **19, 20**) establish π-π interactions with W386 in WT (Supplementary Figure 5). Due to their different shapes, each ligand engages distinct H-bonds patterns (Supplementary Table 3). This network in compounds **13** and **20** involves residues G521, M528, and C530, while **9** and **21** persistently H-bond either to L346, similarly to AZD, or to E419 and G420 (Supplementary Table 3). These results show that the selected compounds can either bind in LBC (**9** and **21**) or interact with H11-12 loop (**13** and **20**).

Conversely, **19** is the only ligand firmly anchored to E419 and L346 (Supplementary Table 3), at tract H-bonding to K529, similarly to AZD in complex with to Y537S (Figure 6). This H-bonding motif in our previous paper was indicated as an essential signature of drug-efficacy. Nevertheless, **19** forms a set of low-persistent H-bonds, underlying its high mobility and the need for further optimization in order to improve its efficacy. Surprisingly, compound **19** establishes a well-defined and stable H-bond network only in one LBCs of WT ERα (Supplementary Table 3).

Next, we inspected how the active drugs counteract the H-bond network responsible of the ERα agonist-like conformation induced by mERα (Supplementary Table 4, Figure 6). A decrease of the E380-Y537S interaction, previously indicated as structural signature of an intrinsic ERα activation, occurs with all ligands, even if this is less effective than upon FULV or AZD binding. What is more, E380, which strongly H-bonds to S537 in the aggressive Y537S ERα variant, upon binding of **9**, **19**, and **21**, rearranges and engages persistent H-bond to L536. Additionally, in the presence of **19**, there is significant change in the H-bond network of L536 backbone. While this latter strongly interacts with backbone of L539 in the presence of all other compounds, **19** weakens it and, as a result, L536 H-bonds to the backbone of L540 (Supplementary Table 4). Remarkably, **19** triggers formation of these H-bonds only in Y537S, but not in WT ERα.

**Figure 7:**
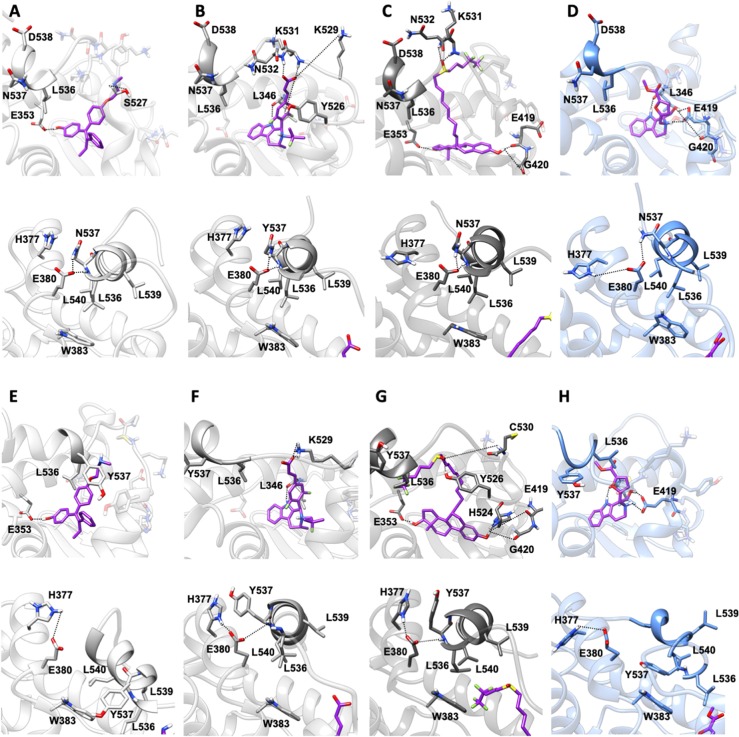
Binding of compound **19** to Y537N and D538G ERα as compared with END, AZD and FULV. Binding to Y537N: **(A)** END; **(B)** AZD; **(C)** FULV; **(D)** Compound **19**. Binding to D538G: **(E)** END; **(F)** AZD; **(G)** FULV; **(H)** Compound **19**. Top panels show their placement in the ligand binding cavity, while bottom panels display a close view of E380 H-bond network induced by each ligand. Inhibitors are shown in licorice with carbon atoms in purple color, while oxygen and nitrogen in red and blue, respectively. Protein is shown in gray new cartoons for END, AZD, FULV, and in blue new cartoons for **19**, respectively.

In both Y537N and D538G variants **19** establishes a H-bond network in the binding cavity and in the H11-12 loop region similarly to Y537S (Supplementary Tables 3, 4, and Figure 7). Hence, **19** exclusively H-bonds to E419 in all mutants, while only in Y537S can establish week H-bond to K529, similarly to AZD.

We have also calculated the binding free energy (ΔG_b_) (Table 1) of the five active compounds to WT, Y537S and, for compound **19**, also to the Y537N, D538G variants. Stunningly, **19** dissociates from one monomer of WT ERα due to the lack of H-bonds, rationalizing its preference toward the pathogenic variants. Instead, its ΔG_b_ is similar in all tested mutants. All other active ligands, but **20**, display a slightly higher affinity for WT ERα, and their ΔG_b_ is slightly larger than that of **19** toward Y537S, most probably because of their larger size. Nonetheless, the tested ligands do not strongly bind to the LBC, as shown by a comparison of the calculated ΔG_b_ compared to that AZD. Thus, even small differences in the position of their H-bonding moieties may result in the different binding poses observed for **20** and **13**. To monitor the impact of the distinct ligands size on ΔG_b_, we also computed the ligand efficiency (LE, Supplementary Table 5), calculated as ΔG_b_ divided by the number of non-hydrogen atoms. LE differences among ligands are smaller than that of ΔG_b_s. **19** presents comparable LE for all mutants tested. Interestingly, compounds **9**, **20**, **21** have a slightly larger LE for Y537S than **19**, suggesting that other features, besides LE or ΔG_b_, may be important for ligand selectivity toward the distinct ERα isoforms.

**Table 1:** Binding free energies (ΔG_b_, kcal/mol) of the ligands **9**, **13**, **19**, **20**, and **21**.

	**WT**		**Y537S**		**Y537N**		**D538G**	
	**A**	**B**	**A**	**B**	**A**	**B**	**A**	**B**
Endoxifen	−51.7 ± 3.6	−46.9 ± 3.3	−45.6 ± 3.5	−45.2 ± 3.5	−43.8 ± 3.3	−49.9 ± 4.3	−47.1 ± 3.4	−50.5 ± 3.6
AZD−9496	−45.2 ± 3.6	−43.9 ± 3.7	−42.9 ± 3.7	−53.7 ± 7.7	−45.1 ± 4.1	−42.9 ± 3.9	−45.1 ± 4.0	−43.7 ± 4.3
Fulvestrant	−69.0 ± 6.4	−66.9 ± 6.2	−65.1 ± 5.1	−76.0 ± 5.6	−68.2 ± 6.1	−66.6 ± 5.7	−63.5 ± 6.0	−69.7 ± 6.0
**9**	−42.7 ± 4.2	−36.7 ± 4.4	−36.9 ± 5.7	−37.3 ± 4.6	/	/	/	/
**13**	−44.8 ± 4.3	−42.6 ± 3.3	−41.4 ± 3.2	−45.2 ± 4.6	/	/	/	/
**19**	−21.3 ± 7.2^*^	−40.9 ± 8.1	−32.3 ± 5.8	−32.3 ± 6.2	−32.9 ± 3.6	−43.0 ± 6.7	−30.8 ± 5.7	−46.8 ± 6.0
**20**	−38.6 ± 3.3	−35.1 ± 2.6	−40.0 ± 2.9	−38.9 ± 5.0	/	/	/	/
**21**	−45.4 ± 3.3	−42.5 ± 3.8	−43.6 ± 3.2	−39.2 ± 3.0	/	/	/	/

*Absolute values of molecular mechanics Generalized bond surface are (MM-GBSA)^†^*.

*^†^Number of atoms/heavy atoms in each ligand*:

*Endoxifen – 55/28*.

*AZD-9496 – 57/32*.

*Fulvestrant – 88/41*.

***9** – 56/29*.

***13** – 51/30*.

***19** – 46/25*.

***20** – 47/27*.

***21** – 50/26*.

* *Ligand exits from the binding pocket*.

### Structural Signatures of (m)ERα Activation/Inactivation

The cross-correlation matrix calculated on the basis of the Pearson correlation coefficients (CC_ij_) was computed to qualitatively identify the linearly coupled motions between couples of residues along the MD trajectory. A simplified version of this matrix, based on the sum of the of correlation scores (CSs) between each structural elements of (m)ERα (Supplementary Figure 2), has been calculated to decrypt the correlation pattern in complex systems (Casalino et al., 2018), among which ERα (Pavlin et al., 2018). In this analysis, a positive/negative score corresponds to a positively (correlated) / negatively (anti-correlated) motion.

**Figure 8:**
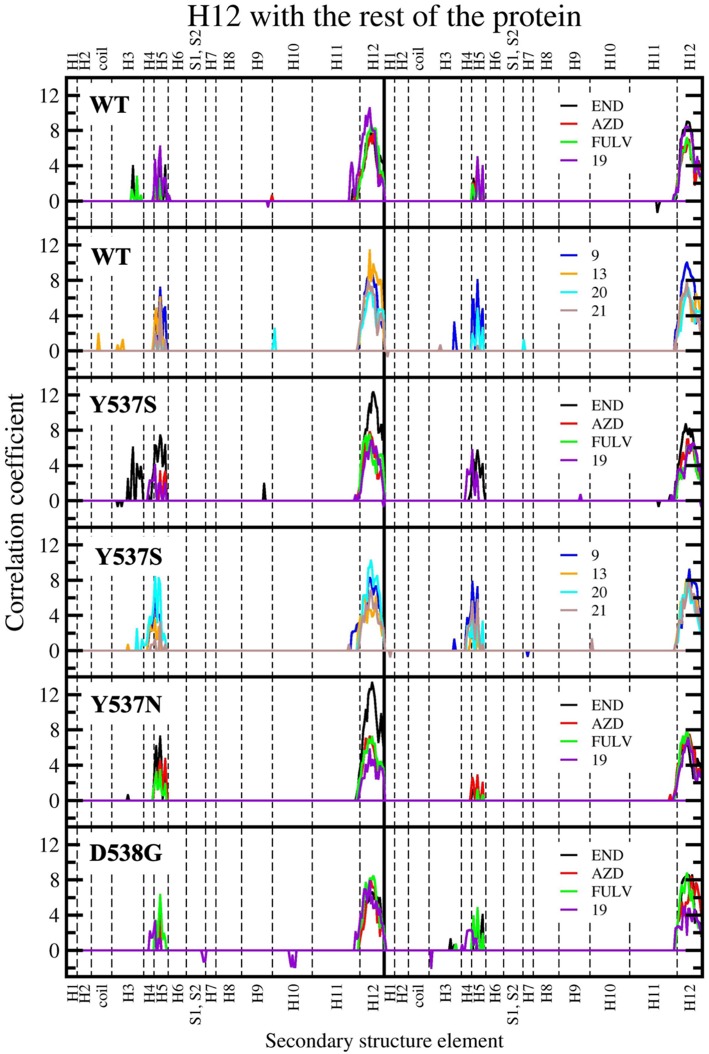
Sum of per-residue cross-correlation coefficients. Left and right columns refer to monomers A and B, respectively. From top to bottom: WT (in complex with END, AZD, FULV, and **19**), WT (with **9**, **13**, **20**, and **21**), Y537S (with END, AZD, FULV, and **19**), Y537S (with **9**, **13**, **20**, and **21**), Y537N, and D538G (in complex with END, AZD, FULV, and **19**). END, AZD, FULV, and compounds **9**, **13**, **19**, **20**, **21** are shown as black, red, green, blue, orange, purple, cyan, and brown lines, respectively.

In our previous study, the presence of a positive correlation score between H12 and H3-H5 was taken as a structural signature of Y537S ERα intrinsic activation. This was persistent upon END binding, while only FULV and, partially, AZD were able to remove it, in line with the proved activity of these SERDs on the Y537S mutant (Fanning et al., 2016). Hence, we also inspected if the ligands differently affect the internal cross-correlation map. All compounds binding to Y537S remove the contacts between H12 and H3, reducing, in most cases, the cross-correlation score in both monomers (Supplementary Figure 6). Moreover, compound **19** decreases these contacts also when binding to Y537N and, to a minor extent, to D538G (Supplementary Figures 7, 8). Conversely, in WT ERα a smaller positive correlation among H5 and H12 can be observed only for (**20** and **21**; Supplementary Figure 9). In order to capture more quantitatively the relative differences among the activity exerted by these ligands we also analyzed how H12 correlates with all other ERα structural elements in the presence of the distinct active compounds. This analysis clearly shows that ligands **9** and **20** present a cross-correlation coupling between H12 and H4-H5 higher or similar to END at least in one Y537S ERα monomer, while **19** effectively reduces this positive correlation in both monomers. This correlation coupling is completely abolished in Y537N and reduced even in D538G (Figure 8), pointing to an activity of **19** also against these mutants, even if to a minor extent.

As a result, **19** appears to reduce the transcriptional activity of Y537S cells thanks to its capability of binding in the LBC of both LBD monomers only in the mERαs, where it establishes H-bonds with E415, L346 and K529, similarly to AZD.

### Kinetic Characterization of Active Compounds

Since increasing evidences pinpoint the dissociation free energy barriers (Δ${\text{G}}_{\text{d}}^{\text{\#}}$) of a ligand from its binding cavity to be strongly entwined with the residence time and, thus, with drugs' efficacy (Magistrato et al., 2017), the observed preferential activity of **19** toward Y537S ERα fostered the investigations of its kinetic properties as compared to those of AZD.

**Figure 9:**
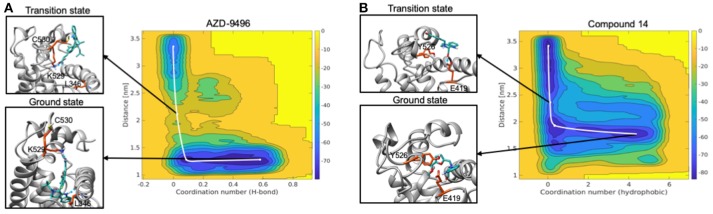
Free energy surface maps from metadynamics simulations for the dissociation of AZD **(A)** and compound **19 (B)** from the ligand binding cavity. On x axis is coordination number (H-bond for AZD and hydrophobic interactions for **19**) and on y axis is COM distance between ligand and receptor. Color scale represents free energy values in kJ/mol. In black squares are encircled structures corresponding to the ligand bound state (ground state), the transition state.

The free energy surface (FES) obtained from MTD simulations inducing the dissociation of AZD from the LBC of Y537S ERα shows a wide minimum at Center of Mass (COM) distance between ligand and protein at ~1.2 nm, which, instead, spans the coordination number (CN) 0.2-0.4. A second, narrower, minimum appears at CN around 0 and COM distance ≥ 2.5 nm. By inspecting two-dimensional FES plots of both CVs one can estimate a Δ${\text{G}}_{\text{d}}^{\text{\#}}$ of 14.1 ± 2.0 kcal/mol for AZD dissociation (Figure 9A, Supplementary Figures 10, 11, and Supplementary Table 6). The main barrier observed for AZD dissociation is due to the breaking of its H-bond interactions between the carboxylic group of AZD with K529 and C530. These, therefore, appear as pivotal residues for increasing the residence time of this drug in the LBC and possibly its efficacy.

On the other hand, FES for the dissociation of **19** reveals a rather wide minimum at CN = 0.2–0.4, lying at higher distance (COM) between ligand and the LBC as compared to AZD (1.5–1.7 nm) (Figure 9B). The second minimum is located in a similar position to that of AZD. In this case, however, the Δ${\text{G}}_{\text{d}}^{\text{\#}}$ is rather small (3.7 ± 1.9 kcal/mol) (Supplementary Figures 10, 11 and Supplementary Table 6) and it is associated to the breaking of the H-bond between the hydroxyl group of the ligand and the E418 residue. This latter, therefore, appears to be a distinctive feature of this ligand.

These simulations pinpoint the most important substituents of the ligand that may contribute to improve the kinetic properties of the drugs, and the residues of the LBC that must be engaged in specific interactions for the discovery of mutant-specific anti-estrogen compounds.

## Discussion

Breast cancer remains the most diagnosed (1 over 8) and the second leading cause of cancer induced mortality in women. The majority (70 %) of BC is hormone dependent and its proliferation relies on the presence of ERα, which has a pro-oncogenic effect in the presence of estrogens. The gold standard treatment in this type of BC is the hormone adjuvant therapy, which either suppresses estrogen production (aromatase inhibitors) or modulates/degrades the ERα (SERMs/SERDs). The prolonged exposure to these therapies, usually administered consecutively for 5–10 years' time-frame, leads to resistance in half of all luminal BCs after 5 years, in spite of the ERα expression (Toy et al., 2017; Fanning et al., 2018; Busonero et al., 2019; Spinello et al., 2019b).

While the genomic profile of inherited and somatic alterations characterizing each type of BC is well-established, the evolution of the BC's genomic landscape under the evolutionary pressure of systemic therapies is not clearly understood. As well as how this landscape impacts on the clinical outcome of endocrine therapies remains poorly characterized and is currently object of intense research efforts. Resistance onset is, in fact, responsible of refractory BCs and of an increased mortality rate. In this worrisome scenario, the therapeutic options to intervene with personalized treatments based on the patients' evolution of the genomic profiles remains a daunting challenge. This has spurred substantial efforts to characterize the phenotype responsible of drug resistance and propose innovative therapeutic options.

Distinct studies indicated that frequent mutations present in the loop connecting H11 and H12 of the LBD trigger the acquisition of an intrinsically active (agonist-like) ERα conformation, even in the absence of E2. This conformation remains even in the presence of SERMs (Fanning et al., 2016; Pavlin et al., 2018). Our recent computational attempt to identify the key common structural traits that drugs should possess in order to effectively fight resistant BCs was the grounding knowledge for the present study. Indeed, here we carried out *in silico* screening on the structural scaffolds of the Y537S, Y537N, and D538G mutants adapted to known mERα degraders (AZD and FULV), seeking for the structural elements able to protrude toward loop connecting H11-H12. This should allow the ligand to counteract the intrinsic and mutant dependent ERα activation (Pavlin et al., 2018).

From a consensus docking study, we selected 17 molecules (Supplementary Figure 3) effectively binding in at least two mutants, among which five resulted to be active on BC cell lines. Some of them were known to be active also on other targets and diseases (Supplementary Table 7). Among these compounds, **19** was selective exclusively toward those expressing Y537S (and to a minor extent to Y537N, D538G) ERα (Figures 2–4). In spite of its ability to block the transcriptional activity of the receptor only in the high μM range, thus requiring further optimization, the structural scaffold of compound **19** encompasses all the motifs required by an active and mutant-specific drug-candidate. Namely, **19** forms number of H-bonds in the ligand binding cavity (L346 and E419) and with K529. Conversely, E380, a key residue involved in the structural transition toward an agonist-like state of the receptor, persistently H-bonds to H377. This is a previously annotated structural feature able to impede the pro-oncogenic effect of resistant phenotypes. Indeed, compound **19**, to the best of our knowledge, is the only mutant specific modulator of ERα transcriptional activity identified so far. However, its ΔG_b_ and Δ${\text{G}}_{\text{d}}^{\text{\#}}$ are remarkably smaller than the parent AZD compound. A detailed comparison among the residues, which optimize these thermodynamics and kinetic properties of the **19** with respect to those of AZD is informative for future knowledge-based drug-design efforts aimed at discovering drug-candidates with superior efficacy.

Since ESR1 mutations are potential clinical biomarkers to guide therapeutic decisions, identification of small molecules able to block proliferation of metastatic tumors expressing one prevalent mERα resistant phenotypes may result in counteracting, preventing and/or delaying their occurrence in early disease stage. In this scenario, our study contributes to move a step forward toward precision and personalized medicine tailored against metastatic and resistant ER+ BCs.

## Data Availability

All datasets generated for this study are included in the manuscript/Supplementary Files.

## Author Contributions

LG and IB performed experiments. MP and AS performed simulations and analysis. IB, SA, SC, and AM designed research and wrote the manuscript.

### Conflict of Interest Statement

The authors declare that the research was conducted in the absence of any commercial or financial relationships that could be construed as a potential conflict of interest.

## Supporting Information

Complementary figures and tables are reported in the supporting information. Structures of the docked compounds before and after molecular dynamics simulation are available upon request to the authors.
