# Supplementary material for: Structural, Thermodynamic, and Kinetic Traits of Antiestrogen-Compounds Selectively Targeting the Y537S Mutant Estrogen Receptor α Transcriptional Activity in Breast Cancer Cell Lines
Source: Front Chem. 2019 Sep 4;7:602. doi: 10.3389/fchem.2019.00602 (PMC6737084; doi:10.3389/fchem.2019.00602)
Supplement: Supplementary file 1 [file Table_1.DOCX]

Supplementary Material

# Supplementary Figures and Tables

## Supplementary Tables

**Supplementary Table 1.** Docking scores of compounds **6**-**22** on the distinct target structures. In the first column is reported the numbering of the compounds in this paper and in second their number code in National Cancer Institute database (NCI ID). In remaining columns (3-8) is reported the docking score (GlideScore) on the distinct structures obtained from our previous Molecular Dynamics (MD) simulations of AZD bound to Y537S and to D538G, FULV bound to Y537S, Y537N and to D538G, respectively. Highlighted in green are the compounds resulted as active in this study. In italics are reported the ligands selected on the basis of the structure-based virtual screening. The other molecules were selected according to a ligands-based virtual screening approach. Columns highlighted in yellow are representing Y537S mutant (m)ERα polymorphism. In the last column is reported similarity of compounds with respect to AZD-9496 based on the virtual screening using ECFP4 and Tanimoto similarity method.

|  | **NCI ID** | **AZD Y537S** | **AZD D538G** | **FULV Y537S** | **FULV Y537N** | **FULV D538G** | **Similarity vs AZD-9496^*^** |
| --- | --- | --- | --- | --- | --- | --- | --- |
| Endoxifen |  | -8.628 | -9.155 | -10.091 | -8.345 | -10.660 |  |
| AZD-9496 |  | -9.204 | -8.076 | -7.211 | -4.658 | -8.788 |  |
| Fulvestrant |  | -8.646 | -9.124 | -12.543 | -10.967 | -14.211 |  |
| **6** | 72135 | -7.229 |  |  |  | -8.595 | 0.110497 (1%) |
| ***7*** | *132526* | *-9.815* | *-10.658* | *-11.304* |  | *-11.253* | *0.038674 (70%)* |
| **8** | 136505 |  |  | -8.556 |  | -7.064 | 0.057895 (20%) |
| **9** | 143691 |  |  | -7.551 |  | -9.539 | 0.084656 (2%) |
| ***10*** | *169656* |  |  |  |  | *-11.612* | 0.067901 (10%) |
| **11** | 169705 |  |  | -7.357 | -7.463 |  | *0.060000 (20%)* |
| **12** | 229519 |  |  | -7.558 |  | -8.970 | 0.105882 (1%) |
| **13** | 321151 |  |  |  | -8.456 | -10.000 | 0.045455 (50%) |
| ***14*** | *351372* | *-9.536* |  |  |  |  | 0.062147 (15%) |
| ***15*** | *610618* | *-9.126* |  | *-11.673* |  |  | 0.053763 (30%) |
| **16** | 629709 | -6.929 |  | -7.510 |  |  | 0.079096 (5%) |
| **17** | 633547 |  |  | -8.176 |  | -6.753 | 0.094444 (1%) |
| **18** | 634123 |  |  | -8.543 | -7.151 | -8.492 | 0.152439 (1%) |
| **19** | 647384 | -8.348 | -7.741 |  |  |  | 0.091429 (1%) |
| **20** | 647388 |  | -8.737 | -7.470 |  | -7.288 | 0.084746 (2%) |
| **21** | 679515 | -7.707 | -8.448 |  |  |  | 0.090396 (2%) |
| **22** | 688534 | -8.362 | -8.247 | -8.500 |  |  | 0.078212 (5%) |

^*^The compound with highest similarity score vs AZD-9496 has value of 0.165468. Percentage value in parentheses shows the ranking of the similarity score of the compound with respect to the whole library (*i. e.* 20% means that compound is in top 20% of all compounds).

**Supplementary Table 2.** Oligonucleotide primers used in this study.

| *Gene Name* | *Gene Symbol* | *Primer Sequences* |
| --- | --- | --- |
| Cyclin D1 | *CCND1* | Fw: 5’-ATGAACTACCTGGACCGCTT-3’ |
|  |  | Rv: 5’-CTTAGAGGCCACGAACATGC-3’ |
| Cathepsin D | *CTSD* | Fw: 5’-CCTCCATCCACTGCAAACTG-3’ |
|  |  | Rv: 5’-TTCACGTAGGTGCTGGACTT-3’ |
| c-myc | *MYC* | Fw: 5’-CACAGCAAACCTCCTCACAG-3’ |
|  |  | Rv: 5’-TGCGTAGTTGTGCTGATGTG-3’ |
| Trefoil Factor-1/ps2 | *TFF1* | Fw: 5’-GGGGTCCCCTGGTGCTTCTA-3’ |
|  |  | Rv: 5’-GCGTCAGGATGCAGGCAGAT-3’ |

**Supplementary Table 3.** Hydrogen (H)-bonds between ligand and the receptor.

(a) H-bonds between ligand and protein (ligand is H-bond donor)

|  | L346 – Ligand  (O^…^H-N)  (O^…^H-O) | | E419 – Ligand  (Oe1/Oe2^…^H-N)  (O^...^H-N)  (O^…^H-O)  (Oe1/Oe2^…^H-O) | | G420 – Ligand  (O^…^H-N)  (O^…^H-O) | | G521 – Ligand  (O^…^H-N) | | M528 – Ligand  (O^…^H-O)  (O^…^H-N) | | K529 – Ligand  (O^…^H-N) | | C530 – Ligand  (O^…^H-O)  (O^…^H-N) | |
| --- | --- | --- | --- | --- | --- | --- | --- | --- | --- | --- | --- | --- | --- | --- |
| **WT** | | | | | | | | | | | | | | |
| Endoxifen |  |  |  |  |  |  |  |  |  |  |  |  |  |  |
| AZD-9496 | 83.9  / | 81.8%  / |  |  |  |  |  |  |  |  |  |  |  |  |
| Fulvestrant |  |  |  |  |  |  |  |  |  |  |  |  |  |  |
| **9** |  |  | 88.7%  7.3%  /  / |  |  | 23.0%  / |  |  |  |  |  |  |  |  |
| **13** |  |  |  |  |  |  |  |  | 27.8%  / |  |  |  | 18.3%  / |  |
| **19** |  |  |  |  |  |  |  |  |  |  |  |  |  |  |
| **20** |  |  |  |  |  |  | 59.9% |  |  |  |  |  |  |  |
| **21** | 90.2%  / | 69.5%  / |  |  |  |  |  |  |  |  |  |  |  |  |
| **Y537S** | | | | | | | | | | | | | | |
| Endoxifen |  |  |  |  |  |  |  |  |  |  |  |  |  |  |
| AZD-9496 | 74.8%  / | 79.6%  / |  |  |  |  |  |  |  |  |  |  |  |  |
| Fulvestrant |  |  |  |  |  |  |  |  |  |  |  |  |  |  |
| **9** |  |  |  |  |  | 42.8%  / |  | 4.2% | /  35.8% |  | 2.3% |  |  |  |
| **13** |  |  |  | /  /  3.2%  / |  |  |  |  | 15.5%  / |  | 18.3% |  |  |  |
| **19** | 60.4%  / | 42.3%  19.2% | 10.3%  /  18.6%  12.1% | 12.1%  /  4.6%  56.8% |  |  |  | 4.3% |  |  |  |  |  |  |
| **20** |  |  |  |  |  |  |  |  |  |  |  |  |  |  |
| **21** | 91.3%  / | 91.6%  / |  |  |  |  |  |  |  |  |  |  |  |  |
| **Y537N** | | | | | | | | | | | | | | |
| Endoxifen |  |  |  |  |  |  |  |  |  |  |  |  |  |  |
| AZD-9496 | 80.6%  / | 74.1%  / |  |  |  |  |  |  |  |  |  |  |  |  |
| Fulvestrant |  |  | /  /  91.7%  / | /  /  56.3%  / |  | /  27.0% |  |  |  |  |  |  |  |  |
| **19** | 85.8%  / | 69.3%  / |  | 94.1%  /  /  67.7% | /  9.6% |  |  |  |  |  |  |  |  |  |
| **D538G** | | | | | | | | | | | | | | |
| Endoxifen |  |  |  |  |  |  |  |  |  |  |  |  |  |  |
| AZD-9496 | 71.2%  / | 72.4%  / |  |  |  |  |  |  |  |  |  |  |  |  |
| Fulvestrant |  |  | * | /  /  79.2%  / |  |  | /  6.9% |  |  |  |  |  |  |  |
| **19** | 28.6%  1.8% | 62.1%  / | /  49.6%  4.0%  / | 44.0%  /  3.7%  46.5% |  |  |  |  |  |  |  |  | /  4.5% |  |

*Here fulvestrant forms H-bond with H524 (ND1^…^H-O) with occurrence of 94.8%.

(b) H-bonds between ligand and protein (ligand is H-bond acceptor).

|  | Ligand – W383  (O^…^H-Ne1) | | Ligand – E419  (O^…^H-N) | | Ligand – K529  (O^…^HZ*-NZ) | | Ligand – C530  (O^…^H-N) | | Ligand – L536  (O^…^H-N) | | Ligand – S537  (O^…^H-N)  (O^…^H-Og) | |
| --- | --- | --- | --- | --- | --- | --- | --- | --- | --- | --- | --- | --- |
| **WT** | | | | | | | | | | | | |
| Endoxifen |  |  |  |  |  |  |  |  |  |  |  |  |
| AZD-9496 |  |  |  |  | 31.1% | 29.7% |  |  |  |  |  |  |
| Fulvestrant |  |  |  |  |  |  |  |  | 46.3% |  |  |  |
| **9** |  |  |  |  |  |  |  |  |  |  |  |  |
| **13** |  |  |  |  |  |  |  |  |  |  |  |  |
| **19** |  |  |  |  |  |  |  |  |  |  |  |  |
| **20** |  |  |  |  |  |  |  |  |  |  |  |  |
| **21** |  |  |  |  |  |  |  |  |  |  |  |  |
| **Y537S** | | | | | | | | | | | | |
| Endoxifen |  |  |  |  |  |  |  |  |  |  |  |  |
| AZD-9496 |  |  |  |  |  | 32.7% |  | 22.1% |  | 69.2% |  | 36.1%  38.0% |
| Fulvestrant |  |  |  |  |  |  |  |  | 10.0% | 71.6% |  |  |
| **9** |  |  |  |  |  |  |  |  |  |  |  |  |
| **13** |  |  |  |  |  |  |  |  |  |  |  |  |
| **19** |  | 3.1% | 1.4% |  | 15.2% |  |  |  |  |  |  |  |
| **20** |  | 32.1% |  |  |  |  |  | 1.1% |  |  |  |  |
| **21** |  |  |  |  |  |  |  |  |  |  |  |  |
| **Y537N** | | | | | | | | | | | | |
| Endoxifen |  |  |  |  |  |  |  |  |  |  |  |  |
| AZD-9496 |  |  |  |  | 19.0% | 25.3% |  |  |  |  |  |  |
| Fulvestrant |  |  |  |  |  |  |  |  | 46.3% |  |  |  |
| **19** |  |  |  |  |  |  |  |  |  |  |  |  |
| **D538G** | | | | | | | | | | | | |
| Endoxifen |  |  |  |  |  |  |  |  |  |  |  |  |
| AZD-9496 |  |  |  |  | 63.1% | 44.6% |  |  |  |  |  |  |
| Fulvestrant |  |  |  |  |  |  |  | 48.5% |  |  |  |  |
| **19** |  |  |  |  |  |  |  |  |  |  |  |  |

**Supplementary Table 4.** H-bonds between H12 and rest of the estrogen receptor α (ERα) as considered in our previous study.^1^

|  | L536 – L540  (O^…^H-N) | | L536 – L539  (O^…^H-N) | | E380 – L536  (Oe1/Oe2^…^H-N) | | E380 – Mut537  (Oe1/Oe2^…^H-N)  (Oe1/Oe2^…^H-O) | | E380 – H377  (Oe1/Oe2^…^H-Nd1) | |
| --- | --- | --- | --- | --- | --- | --- | --- | --- | --- | --- |
| **WT** | | | | | | | | | | |
| Endoxifen | 81.7% | 73.2% | 7.9% | 2.7% | / | / | 35.0%  / | 7.9%  / | 73.5% | 81.9% |
| AZD-9496 | 84.8% | 76.1% | 13.0% | 7.2% | / | / | 8.4%  / | 26.0%  / | 4.4% | / |
| Fulvestrant | 30.0% | 64.9% | 3.3% | 3.4% | / | / | /  / | 3.0%  / | 63.5% | 82.4% |
| **9** | 68.2% | 80.6% | / | / | / | / | 2.7%  / | 10.2%  / | 93.1% | 72.2% |
| **13** | 68.3% | 53.7% | / | / | / | / | /  / | 14.9%  / | 69.0% | 3.3% |
| **19** | 83.1% | 65.6% | / | / | / | / | 1.7%  / | 9.1%  / | 77.8% | 77.3% |
| **20** | 73.4% | 78.9% | / | / | / | 15.9% | 13.7%  / | 39.9%  / | 49.3% | 27.9% |
| **21** | 79.5% | 5.1% | / | 24.7% | / | / | 15.7%  / | /  / | 68.1% | 62.2% |
| **Y537S** | | | | | | | | | | |
| Endoxifen | 78.5% | 74.8% | 7.1% | 6.2% | / | / | /  45.9% | 35.3%  71.9% | 65.7% | 60.8% |
| AZD-9496 | 82.0% | 23.6% | 13.3% | 4.1% | / | / | 61.5%  87.7% | / | 17.1% | 59.2% |
| Fulvestrant | 81.0% | 42.2% | 13.7% | 36.8% | / | / | 12.4%  37.4% | /  27.2% | 34.2% | 12.3% |
| **9** | 85.4% | 81.6% | / | 33.3% | / | 83.4% | 53.2%  86.8% | 79.3%  95.6% | 8.8% | 11.4% |
| **13** | 79.5% | 84.4% | / | / | / | / | 1.5%  50.2% | 68.4%  93.7% | 70.0% | 12.5% |
| **19** | 45.9% | 83.4% | 58.1% | 17.5% | 94.0% | / | 80.0%  97.2% | 37.3%  85.9% | 60.0% | 35.9% |
| **20** | 80.0% | 86.2% | / | / | / | / | 46.7%  88.8% | 72.0%  96.4% | 29.6% | 3.0% |
| **21** | 84.4% | 81.1% | 16.1% | 20.5% | / | 27.7% | 62.5%  94.1% | 72.3%  96.1% | 43.0% | 1.0% |
| **Y537N** | | | | | | | | | | |
| Endoxifen | 85.5% | 84.0% | 2.3% | 2.8% | / | / | 50.4%  90.3%* | 76.3%  102.4%* | 22.3% | 5.1% |
| AZD-9496 | 83.7% | 81.3% | 3.6% | 3.0% | / | / | 67.5%  98.1%* | 69.7%  102.8%* | 6.4% | 16.1% |
| Fulvestrant | 71.4% | 83.8% | 5.0% | 6.1% | / | / | 26.7%  70.7%* | 54.2%  89.7%* | 22.2% | 11.7% |
| **19** | 81.6% | 80.4% | 21.6% | 16.1% | 22.7% | 7.7% | 20.2%  63.8%* | 3.6%  24.6%* | 46.3% | 25.1% |
| **D538G** | | | | | | | | | | |
| Endoxifen | 71.8% | 85.7% | 2.6% | 4.6% | / | / | /  18.8% | 29.4%  / | 41.4% | 82.4% |
| AZD-9496 | 79.6% | 82.0% | 13.6% | 8.0% | / | / | 10.0%  / | 21.5%  / | 35.3% | 56.0% |
| Fulvestrant | 77.0% | 82.2% | 10.7% | 4.2% | / | / | 7.1%  / | /  / | 69.6% | 83.8% |
| **19** | 76.0% | 55.1% | 20.2% | 12.1% | / | / | 0.7%  17.5% | 0.6%  0.3% | 56.1% | 21.4% |

*This hydrogen bond is formed between Oe1/Oe2^…^H-ND2.

**Supplementary Table 5**. Ligand binding efficiency (LE) to WT, Y537S, Y537N, D538G calculated as the binding free energies (∆G_b_, kcal/mol) of the 5 active compound against Y537S calculated as absolute values of Molecular Mechanics Generalized Bond Surface Area (MM-GBSA) divided by number of heavy atoms in each ligand.^‡^

|  | WT | | Y537S | | Y537N | | D538G | |
| --- | --- | --- | --- | --- | --- | --- | --- | --- |
|  | A | B | A | B | A | B | A | B |
| Endoxifen | -1.85 ± 0.13 | -1.68 ± 0.12 | 1.63 ± 0.13 | -1.61 ± 0.13 | -1.56 ± 0.12 | -1.78 ± 0.15 | -1.68 ± 0.12 | -1.80 ± 0.13 |
| AZD-9496 | -1.41 ± 0.11 | -1.37 ± 0.12 | -1.34 ± 0.12 | -1.68 ± 0.24 | -1.41 ± 0.13 | -1.34 ± 0.12 | -1.41± 0.13 | -1.37 ± 0.13 |
| Fulvestrant | -1.68 ± 0.16 | -1.63 ± 0.15 | -1.59 ± 0.12 | -1.85 ± 0.14 | -1.66 ± 0.15 | -1.62 ± 0.14 | -1.55 ± 0.15 | -1.70 ± 0.15 |
| **9** | -1.47 ± 0.15 | -1.27 ± 0.15 | -1.27 ± 0.20 | -1.29 ± 0.16 | / | / | / | / |
| **13** | -1.49 ± 0.14 | -1.42 ± 0.11 | -1.38 ± 0.11 | -1.51 ± 0.15 | / | / | / | / |
| **19** | -0.85 ± 0.29^*^ | -1.63 ± 0.32 | -1.29 ± 0.23 | -1.29 ± 0.25 | -1.32 ± 0.14 | -1.72 ± 0.27 | -1.23 ± 0.23 | -1.87 ± 0.24 |
| **20** | -1.43 ± 0.12 | -1.30 ± 0.10 | -1.48 ± 0.11 | -1.44 ± 0.19 | / | / | / | / |
| **21** | -1.75 ± 0.13 | -1.64 ± 0,14 | -1.68 ± 0.12 | -1.51 ± 0.12 | / | / | / | / |

^‡^Number of atoms/heavy atoms in each ligand:

Endoxifen – 55/28

AZD-9496 – 57/32

Fulvestrant – 88/41
**9** – 56/29

**13** – 51/30

**19** – 46/25

**20** – 47/27

**21** – 50/26

***19** goes outside of the LBC in this case.

**Supplementary Table 6.** Dissociation free energy barriers (∆G_b_^#^; kcal/mol) for change in coordination number (CN) and center of mass (COM) distance (nm) from metadynamics (MTD) simulations for dissociation of AZD and **19** from Ligand Binding Cavity (LBC) of ERα Y537S. Results from distinct replicas of the simulations are listed.

|  | AZD | | **19** | |
| --- | --- | --- | --- | --- |
|  | Coordination number | COM distance | Coordination number | COM distance |
| Replica 1 | 1.92 | 16.02 | 1.51 | 4.85 |
| Replica 2 | 1.43 | 12.06 | 1.63 | 1.57 |
| Replica 3 | 0.93 | 14.36 | 4.02 | 4.68 |
| Average | 1.4 ± 0.5 | 14.1 ± 2.0 | 2.4 ± 1.41 | 3.7 ± 1.9 |

**Supplementary Table 7.** Previously known activity of selected compounds. as reported in the NCI database (<https://dtp.cancer.gov/dtpstandard/dwindex/index.jsp>, date of access: 25/07/2019).

| Compound | Trivial name | Known activity |
| --- | --- | --- |
| **9** | Biriperone | Ligand for dopamine receptors D1, D2 and D3, and 5-hydroxytryptamine receptor 2A.^a^ |
| **13** |  | Structural similarity with ormeloxifene, anti-estrogen contraceptive drug. |
| **19** |  | Activity against WT ER+ BC in high concentrations.^b^ |
| **20** |  | No activity againsy WT ER+ BC.^c^ |
| **21** |  | Activity against WT ERER+ BC in high concentrations.^d^ |

^a^<https://pubchem.ncbi.nlm.nih.gov/compound/68663#section=BioAssay-Results> date of access 31/07/2019.

^b^<https://dtp.cancer.gov/services/nci60data/colordoseresponse/pdf/647384> date of access 31/07/2019.

^c^<https://dtp.cancer.gov/services/nci60data/colordoseresponse/pdf/647388> date of access 31/07/2019.

^d^<https://dtp.cancer.gov/services/nci60data/colordoseresponse/pdf/679515> date of access 31/07/2019.

## Supplementary Figures


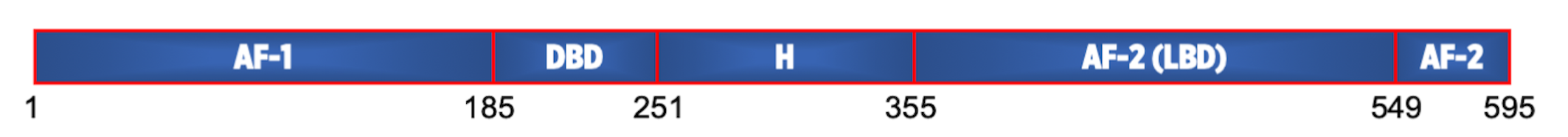


**Supplementary Figure 1.** Multi-domain architecture of one monomer of ERα: Activation function domains, AF-1 and AF-2, DNA-binding domain (DBD), hinge (H) and ligand-binding domain (LBD; which is also part of AF-2).


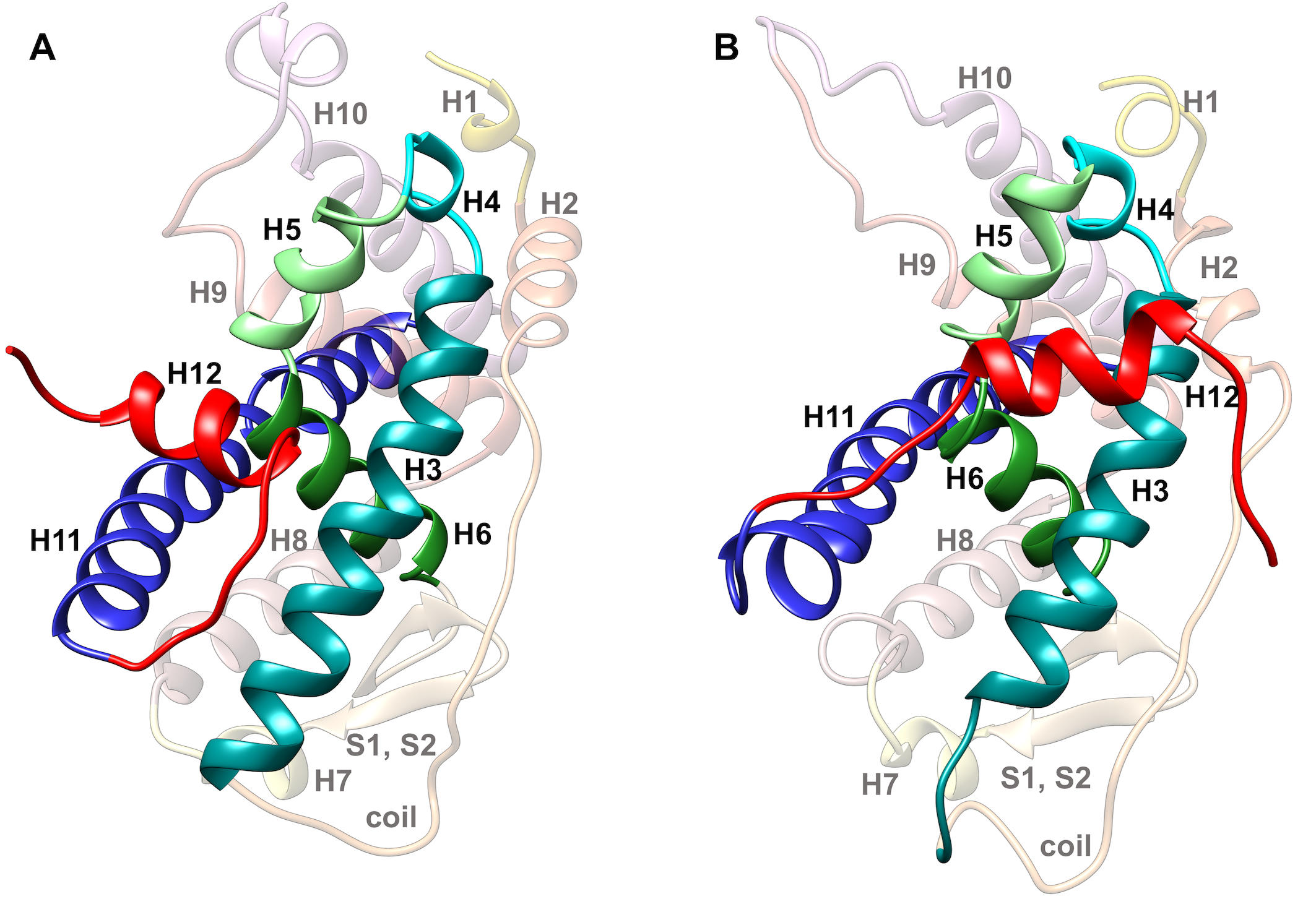


**Supplementary Figure 2**. Structural organization of ERα LBD monomer divided by helices and loops in agonist **(A)** and antagonist **(B)** conformation. The helices label comprises both residues forming helices and those located on the loops connecting them. Namely, H1: residues 303-311 (yellow); H2: residues 312-322 (coral); coil2-3: residues 323-338 (sandy brown); H3: residues 339-363 (dark cyan); H4: residues 364-371 (cyan); H5: residues 372-382 (light green); H6: residues 383-396 (forest green); S1, S2: residues 397-411 (tan); H7: residues 412-419 (khaki); H8: residues 420-439 (rosy brown); H9: residues 440-463 (salmon); H10: residues 464-494 (plum); H11: residues 495-531 (blue); H12: 532-550 (red). Helices interacting with H12 are shown as solid ribbons, while the others are shown as transparent ribbons.


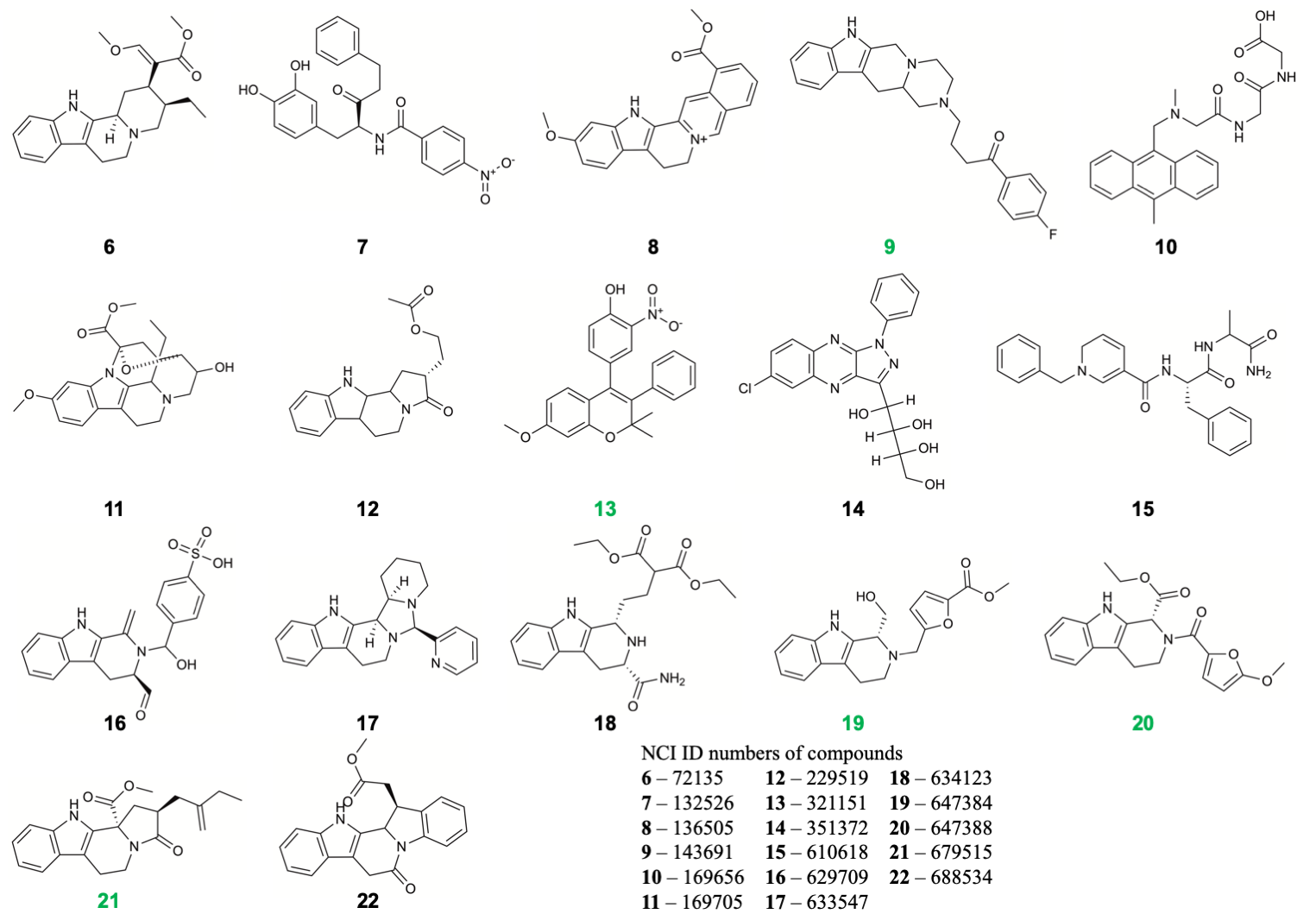


**Supplementary Figure 3.** Sketch of best ranked 17 molecules extracted from the consensus-induced-fit docking strategy employed in this work. Number of the molecule along with their NCI ID number is reported in bold. In green are indicated the molecules, which resulted to be active in cell viability assays.


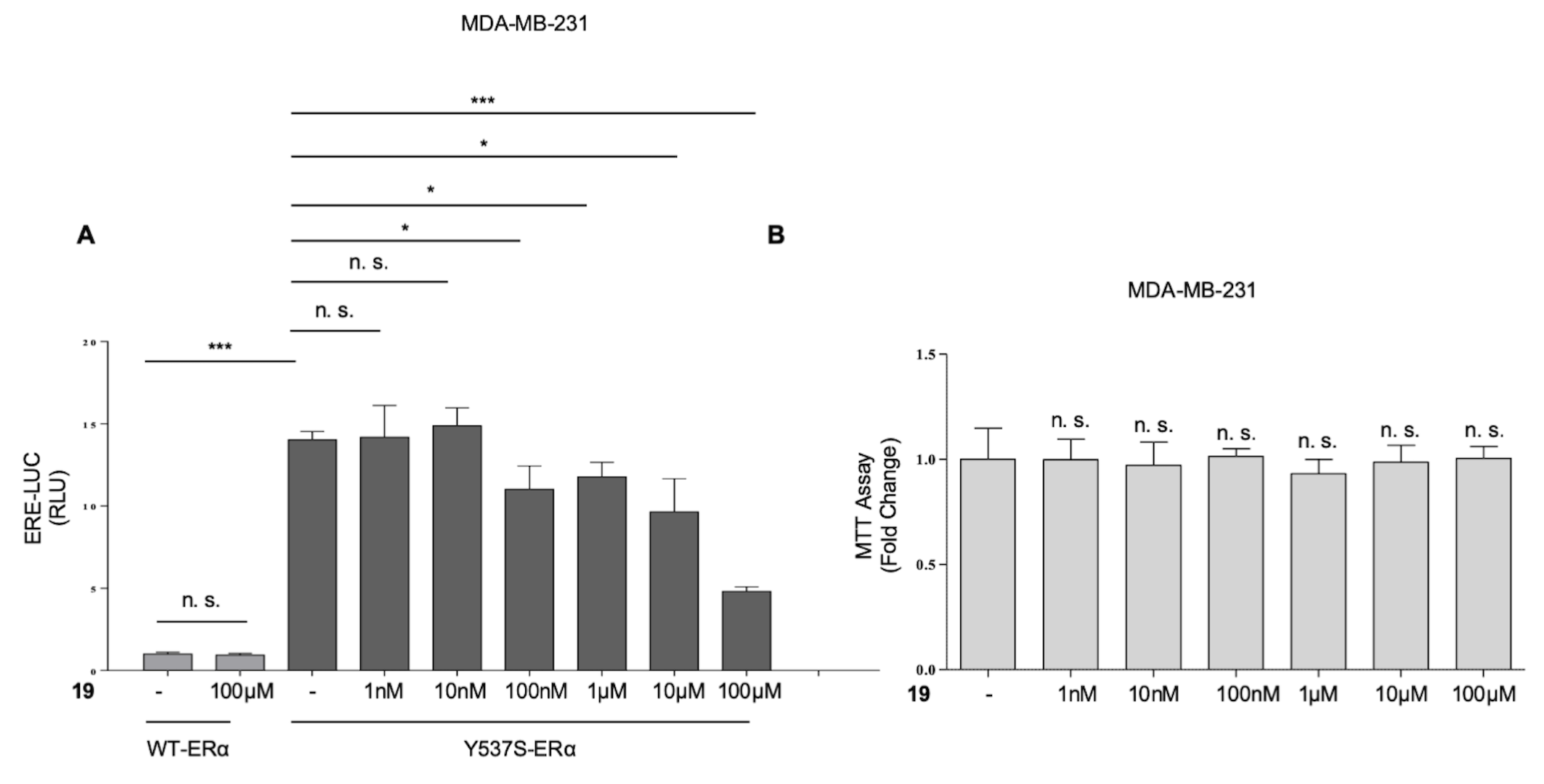


**Supplementary Figure 4.** **A.** Estrogen Receptor transactivation experiments were conducted in ER-negative human MDA-MB-231 breast cancer cells transfected with YFP-WT or YFP-Y537S-ERα vectors plus XETL and treated cells with compound **19** at several doses (from 1 nM to 100 µM). **B.** MTT cell viability assay in MDA-MB-231 breast cancer treated as indicated for 24 hours.


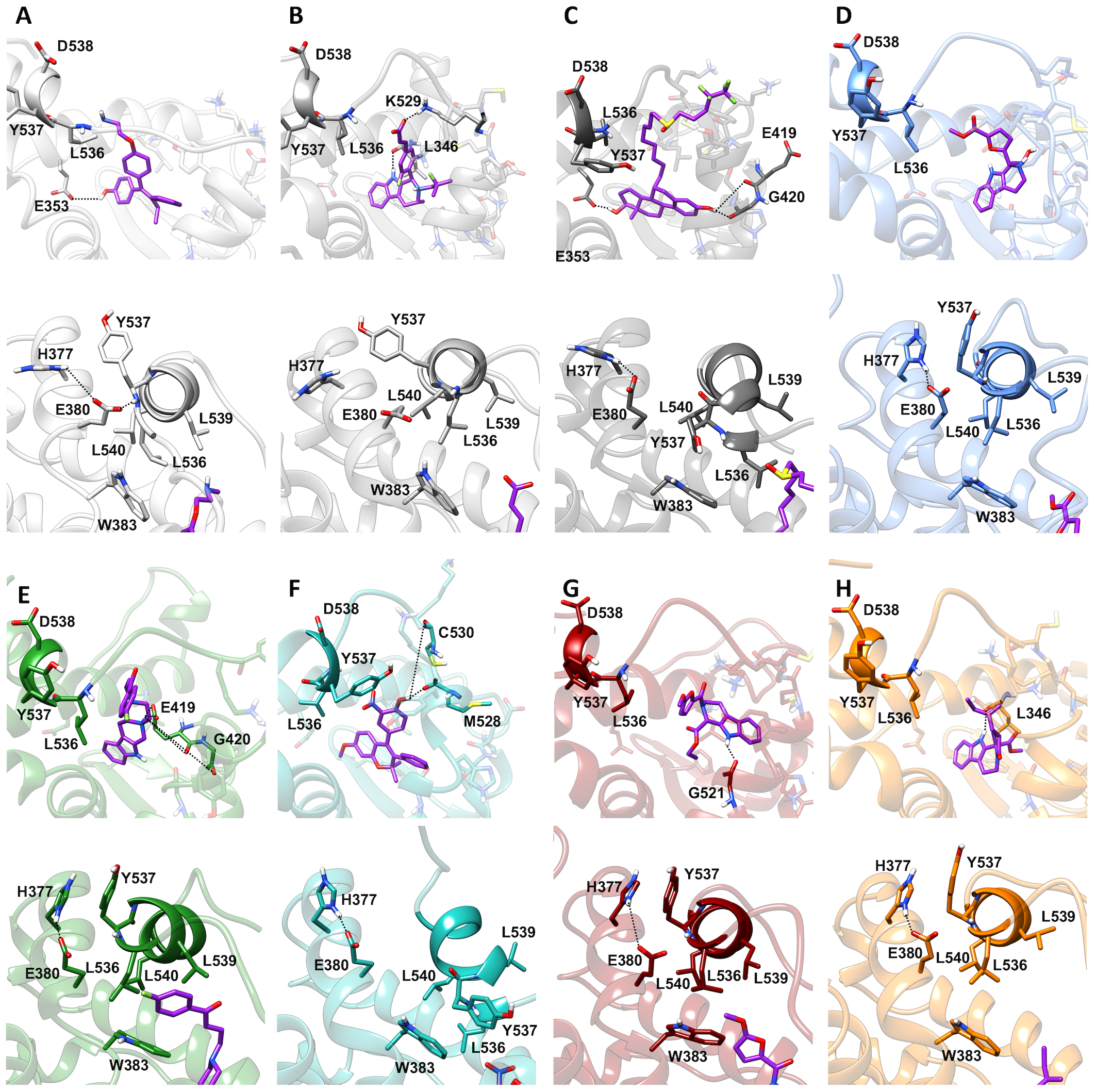


**Supplementary Figure 5.** Binding of the 5 active molecules to WT ERα as compared with END, AZD and FULV. **(A)** END; **(B)** AZD; **(C)** FULV; **(D)** compound **19**; **(E)** **9**; **(F)** **13**; **(G)** **20**; **(H)** **21**. Top panel shows their placement in the ligand-binding cavity and bottom panel shows close view of E380 H-bond network rearrangement as induced by each ligand. Inhibitors are shown in licorice with carbon atoms in purple color, while oxygen and nitrogen in red. Protein is shown as new cartoons in gray color for END, AZD, FULV, and blue, green, light green, purple, orange color for **19**, **9**, **13**, **20**, **21**, respectively.


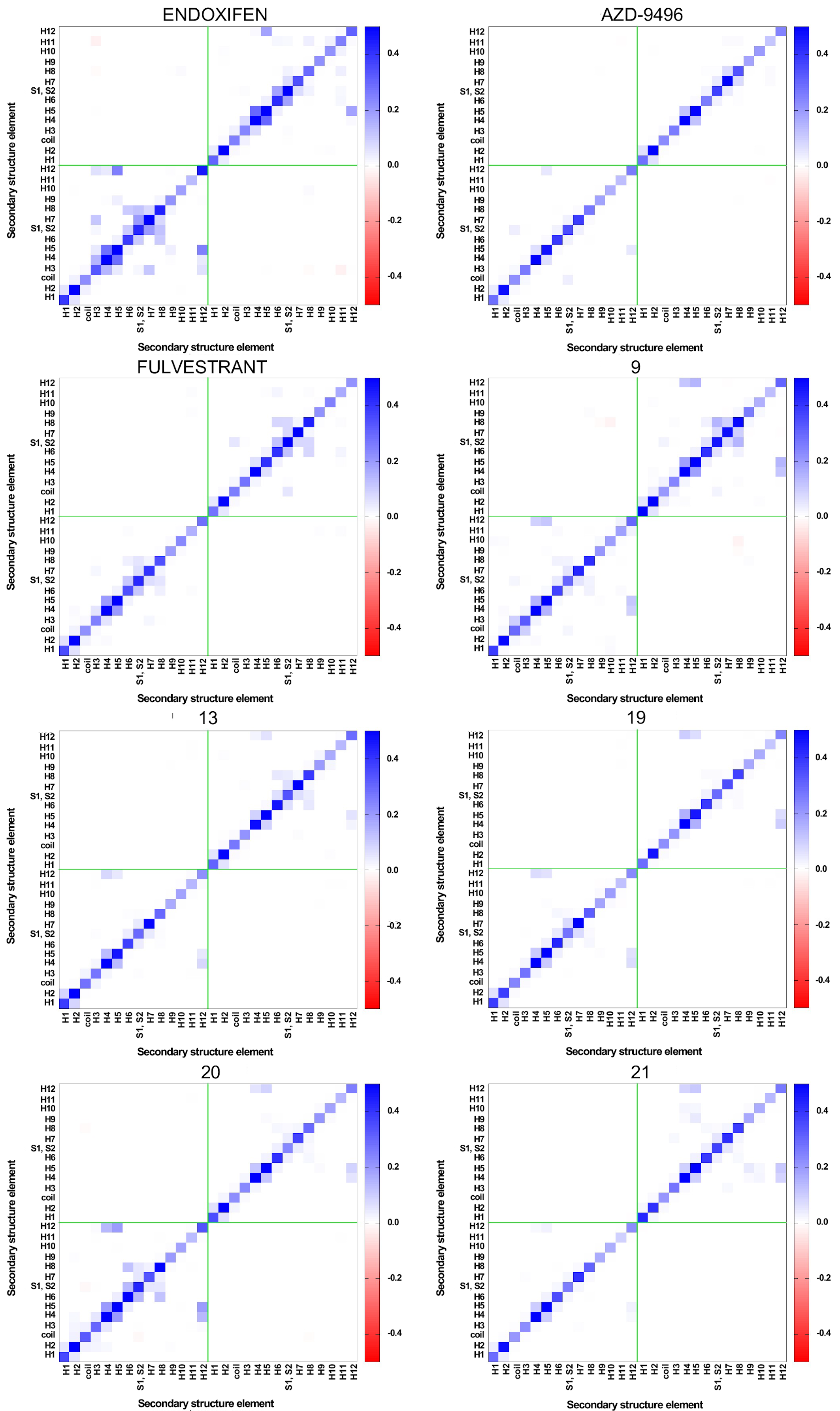


**Supplementary Figure 6.** Cross-correlation maps of 14 regions, as defined in Figure S2 for END, AZD, FULV and compounds **9**, **13**, **19**, **20**, and **21** on Y537S ERα. The cross-correlation coefficients, calculated as the sum of the cross-correlation coefficients (with a correlation score ≥ or ≤ than 0.6 and −0.6) of the residues belonging to two regions considered. Cross-correlation scores are reported in the range from −0.5 to 0.5 for clarity reasons. In this case we report the cross-correlation density in which the score is normalized by the product of number of residues contributing to it after filtering the scores below -0.6 and above 0.6. Blue and red colors account for positive and negative correlation, respectively.


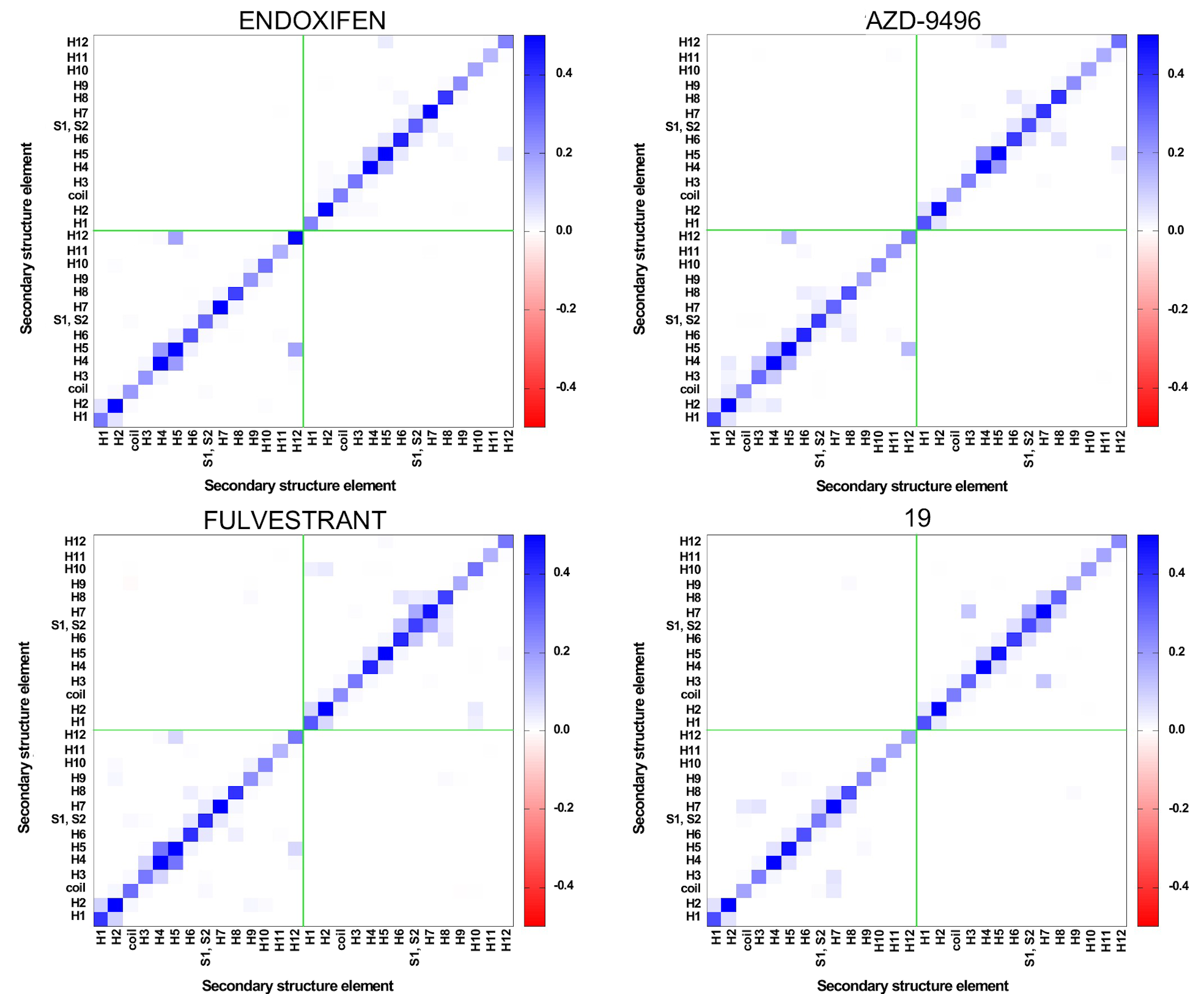


**Supplementary Figure 7.** Cross-correlation maps of 14 regions, as defined in Figure S2 for END, AZD, FULV and compound **19** on D538G ERα. The cross-correlation coefficients, calculated as the sum of the cross-correlation coefficients (with a correlation score ≥ or ≤ than 0.6 and −0.6) of the residues belonging to two regions considered. Cross-correlation scores are reported in the range from −0.5 to 0.5 for clarity reasons. In this case, we report the cross-correlation density in which the score is normalized by the product of the number of residues contributing to it after filtering the scores below -0.6 and above 0.6. Blue and red colors account for positive and negative correlation, respectively.


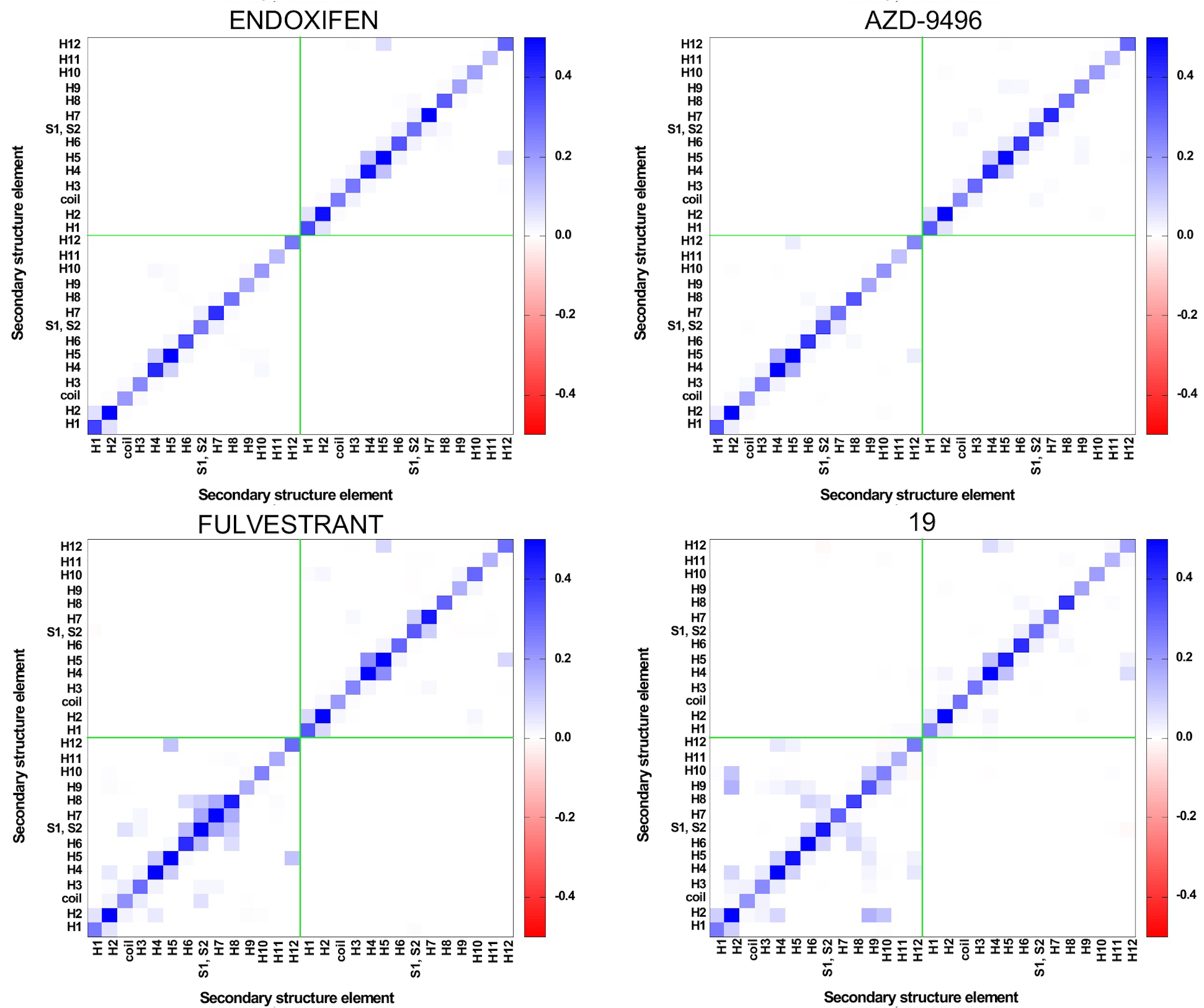


**Supplementary Figure 8.** Cross-correlation maps of 14 regions, as defined in Figure S2 for END, AZD, FULV and compound **19** on D538G ERα. The cross-correlation coefficients, calculated as the sum of the cross-correlation coefficients (with a correlation score ≥ or ≤ than 0.6 and −0.6) of the residues belonging to two regions considered. Cross-correlation scores are reported in the range from −0.5 to 0.5 for clarity reasons. In this case we report the cross-correlation density in which the score is normalized by the product number of residues contributing to it after filtering below -0.6 and above 0.6. Blue and red colors account for positive and negative correlation, respectively.


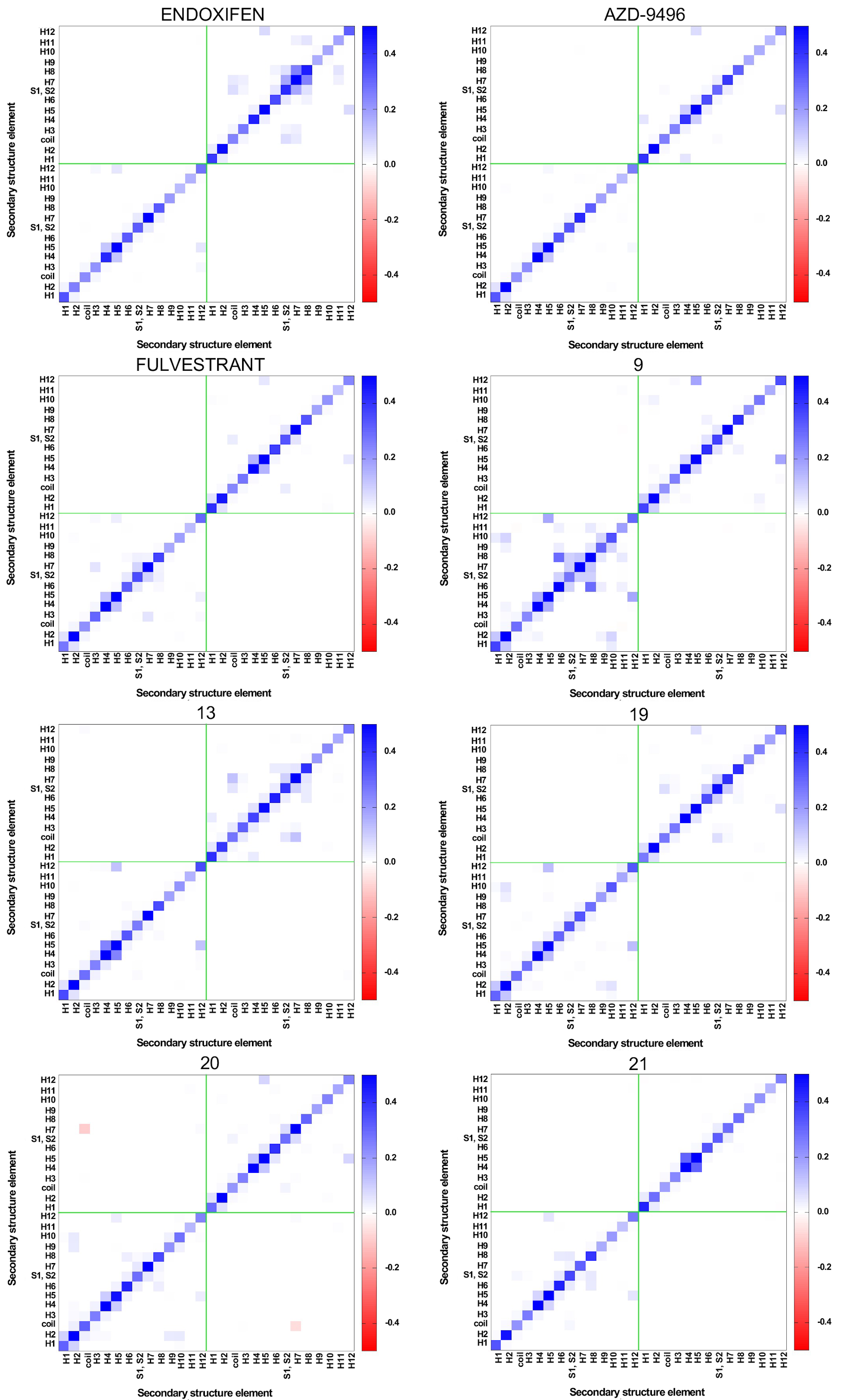


**Supplementary Figure 9.** Cross-correlation maps of 14 regions, as defined in Figure S2 for END, AZD, FULV and compounds **9**, **13**, **19**, **20**, and **21** on wild type ERα. The cross-correlation coefficients, calculated as the sum of the cross-correlation coefficients (with a correlation score ≥ or ≤ than 0.6 and −0.6) of the residues belonging to two regions considered. Cross-correlation scores are reported in the range from −0.5 to 0.5 for clarity reasons. In this case we report the cross-correlation density in which the score is normalized by the product of the number of residues contributing to it after filtering the scores below -0.6 and above 0.6. Blue and red colors account for positive and negative correlation, respectively.

**
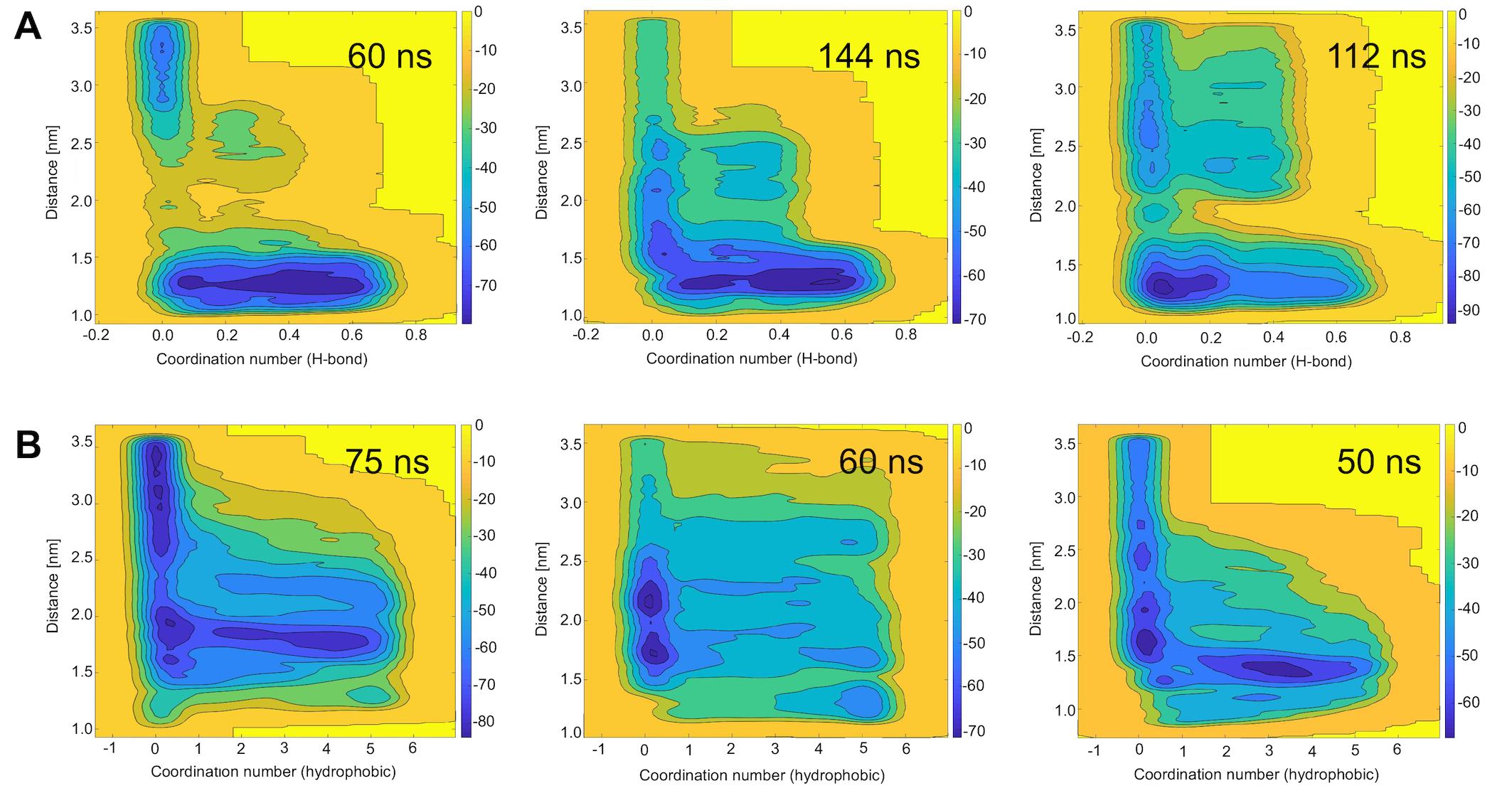
Supplementary Figure 10.** Free energy surface maps from metadynamics simulations (3 replicas for each system) for dissociation of AZD **(A)** and **19 (B)** from LBC. On x-axis is coordination number (H-bond for AZD and hydrophobic interactions for **19**) and on y axis is COM distance between ligand and receptor. Color scale represents the free energy values in kJ/mol. Length of the simulation is denoted on the plot.


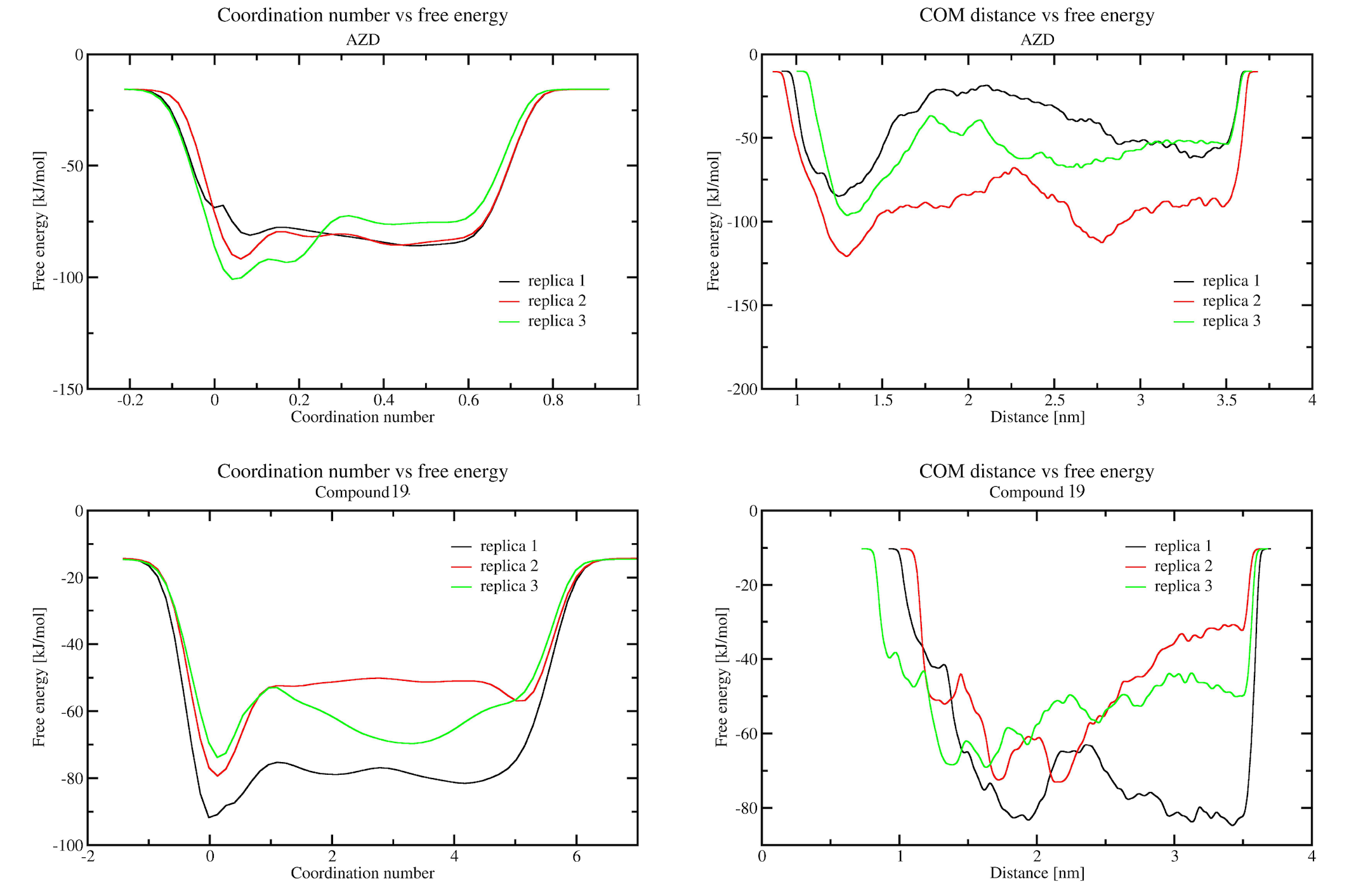


**Supplementary Figure 11.** Two-dimensional maps from metadynamics simulations for ligand dissociation from ERα Y537S LBC. Top left: AZD, coordination number vs free energy; top right: AZD, COM distance vs free energy; bottom left: **19**, coordination number vs free energy; bottom right: **19**, COM distance vs free energy.

## Reference

1. Pavlin, M.; Spinello, A.; Pennati, M.; Zaffaroni, N.; Gobbi, S.; Bisi, A.; Colombo, G.; Magistrato, A., A Computational Assay of Estrogen Receptor alpha Antagonists Reveals the Key Common Structural Traits of Drugs Effectively Fighting Refractory Breast Cancers. *Sci Rep* **2018**, 8, 649.
